# Allosteric GABA_A_ Receptor Modulators—A Review on the Most Recent Heterocyclic Chemotypes and Their Synthetic Accessibility †

**DOI:** 10.3390/molecules25040999

**Published:** 2020-02-24

**Authors:** Blanca Angelica Vega Alanis, Maria Teresa Iorio, Luca L. Silva, Konstantina Bampali, Margot Ernst, Michael Schnürch, Marko D. Mihovilovic

**Affiliations:** 1 Institute of Applied Synthetic Chemistry, TU Wien, Getreidemarkt 9/193, 1060 Vienna, Austria; blanca.alanis@tuwien.ac.at (B.A.V.A.); maria.iorio@tuwien.ac.at (M.T.I.); marko.mihovilovic@tuwien.ac.at (M.D.M.); 2 Department of Anesthesiology and Intensive Care Medicine, Charité–Universitätsmedizin, Charitéplatz 1, 10117 Berlin, Germany; luca@silva.im; 3 Department of Molecular Neurosciences, Center for Brain Research, Medical University of Vienna, Spitalgasse 4, 1090 Vienna, Austria; konstantina.bampali@meduniwien.ac.at

**Keywords:** GABA_A_ modulators, allosteric modulators, heterocyclic modulators, heterocyclic synthesis, nitrogen heterocycles

## Abstract

GABA_A_ receptor modulators are structurally almost as diverse as their target protein. A plethora of heterocyclic scaffolds has been described as modulating this extremely important receptor family. Some made it into clinical trials and, even on the market, some were dismissed. This review focuses on the synthetic accessibility and potential for library synthesis of GABA_A_ receptor modulators containing at least one heterocyclic scaffold, which were disclosed within the last 10 years.

## 1. Introduction

Heterocycles are an extremely diverse compound class since any cyclic system in which at least one carbon is exchanged for any other atom belongs to it. Consequently, they occur abundantly in nature, where they exhibit important functions. For example, the purine and pyrimidine bases in DNA, are heterocyclic, or the amino acids proline, histidine, and tryptophan contain heterocyclic motifs. Naturally, many heterocyclic scaffolds can be found in biologically active natural products such as coniine, atropine, tetrodotoxin, etc. This list could be extended *ad infinitum*. Hence, it is not surprising that drug development also makes excessive use of heterocyclic scaffolds. Their use enabled us to explore and expand the drug-like chemical space. Some privileged structures contain heterocyclic moieties, which are most frequently nitrogen, oxygen, and sulfur. Heterocycles are useful structures to optimize ADME/toxicity features of drug candidates, such as solubility, lipophilicity, or their hydrogen bond donor/acceptor properties. The presence of heterocyclic moieties in recently developed drugs has increased due to advances in synthetic chemistry such as the development of cross-coupling or hetero-coupling reactions, which allow synthetic accessibility to molecules containing functionalized heterocycles. Additionally, some heterocyclic drug compounds have been developed to be inspired by natural product isolates [1].

Even though many small synthetic organic molecules with high medicinal potential contain heterocyclic rings, the range of easily accessible and suitably functionalized heterocyclic building blocks is surprisingly limited, and the construction of even a small array of relevant heterocyclic compounds is far from trivial. Heterocyclic chemistry, therefore, continues to attract the attention of medicinal and synthetic chemists. The development of novel methodologies allowing for efficient access to heterocycles is still highly beneficial [2]. In particular, synthesis of heterocyclic scaffolds has to implement novel reaction technologies for expanding the chemical space of biologically-active molecules such as biocatalytic methods [3], electrochemical methods, flow chemistry [4], and photochemistry [5] to expedite medicinal chemistry programs [6,7].

Within this review, heterocyclic compounds acting as GABA_A_ receptor modulators will be revised. First, an introduction to this specific receptor family and the biological responses related to modulation will be provided. Subsequently, synthetic methods, which have been disclosed in the last 10 years to access various heterocyclic GABA_A_ receptor modulators, will be highlighted. The selection criteria for the examples of compounds included was mainly based on synthetic originality and versatility, and not on any specific pharmacological property. Scaffolds are arranged according to their heterocyclic motif rather than according to their activity profile or binding site.

### 1.1. The Receptor Family

**Figure 1:**
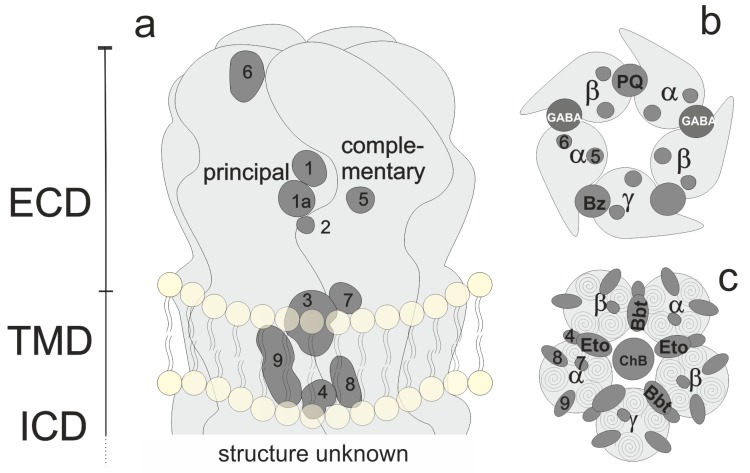
Overview of pentamer structure and localization of orthosteric and allosteric binding sites. (**a**): Generic subunit interface, featuring ECD, TMD, and lower TMD. (**b**) View of the ECD. GABA sites, the benzodiazepine (BZD) site, and the modulatory pyrazoloquinolinone (PQ) site are shown explicitly. (**c**) View of the TMD of a canonical receptor with etomidate (Eto) and barbiturate (Bbt) sites shown explicitly.

Among the mammalian ligand-gated ion channels, the GABA_A_ receptor family comprises the largest family with subunits encoded by 19 different genes. Some of these undergo alternative splicing, and, thereby, increase the variety [8,9]. Their endogenous ligand known as the gamma-aminobutyric acid (GABA) has been established as the main inhibitory neurotransmitter in the central nervous system [10]. These chloride channels are homopentamers or heteropentamers, whereby the subunits share a 30–80% sequence identity [11]. It is generally accepted (though not proven) and suggested by the bulk of experimental evidence that the majority of GABA_A_ receptors in the adult brain are pentamers with the subunit composition two α1, two β2, or β3 and one γ2 subunits, i.e., (α1)_2_(β2 or β3)_2_(γ2)_1_ [9]. The subunit arrangement of this type of receptor is depicted in Figure 1. Each subunit is defined by its position in the pentamer and by its interfaces perceived from the extracellular side, going into counter-clockwise directions. A principal (or plus, +) face is in contact with the next complementary (or minus, −) face, i.e., the canonical orthosteric β+/α− interface. 

For heteromeric receptors of different compositions (such as αβ or αβδ combinations), the arrangements are debated controversially and have not yet been defined conclusively [12,13]. The number of pentameric assemblies that exist has also not been determined [9]. Figure 1 depicts a generic subunit interface, which features sites known or proposed at and near a subunit interface. Regions 1 and 1a form the extracellular domain (ECD) interface site which harbors, e.g., GABA or benzodiazepine sites (see panel b). Region 2 is a cation site while region 3 corresponds with the etomidate or barbiturate sites in the upper transmembrane domain (TMD) (see panel c), while region 4 at the lower TMD is the site where positive modulatory steroids have been observed [14]. Sites more remote to the interface (intrasubunit-sites and lipid-associated sites) for which ligands are confirmed include site 9 [14] for negative modulatory steroids, and tentative sites in positions 5, 6, 7, and 8 [15]. One α subunit shows the remaining sites with the numbers to illustrate their approximate localization from this perspective.

For the major receptor subtypes that have been observed in the mammalian central nervous system, subtype-selective targeting to address specific circuitry and physiology selectively has been attempted and is still an ongoing major direction of ligand development [16]. Knowledge of binding sites is crucial for subtype-selective targeting so that pockets that feature sequence differences can be taken into consideration as discussed for some relevant examples below.

### 1.2. Allosteric Binding Sites

GABA_A_ receptors are targets of many heavily used pharmaceuticals such as benzodiazepine-based sedatives, hypnotics, and anxiolytics, barbiturate-based anticonvulsants, and general anesthetics of a broad range of chemotypes [16,17]. Nearly all pharmaceuticals and a wide range of toxins that target members of the GABA_A_ receptor family do not interact with the binding site for the physiological agonist (GABA) as orthosteric ligands, but rather with one or several allosteric-binding sites [12,15,17]. Figure 1 provides an overview of the described allosteric sites.

Of the sites depicted in Figure 1, some display a high degree of variability, while others are fairly conserved throughout the family [15]. The extracellular interfaces have large variable pocket-forming regions and are, thus, particularly suited for the development of selective agents. The ECD α+/γ− interface features potentially 18 subtypes (six α and three γ subunit isoforms) and is the major site of action of high-affinity benzodiazepine effects [18].

Since their serendipitous discovery and the introduction of chlordiazepoxide (Librium^®^) in 1960, benzodiazepines have spurred enormous efforts to develop benzodiazepine site ligands with further improved properties compared to classical benzodiazepines. Dozens of chemotypes, among them β-carbolines, pyrazoloquinolinones, and imidazopyridines, have been identified as high-affinity ligands of benzodiazepine binding sites. The exact number of high and low-affinity benzodiazepine binding sites is still not completely defined, and even high affinity sites that are not localized to the canonical α+/γ− interfaces have been described [18]. 

Due to the high homology of the α+/β− interfaces with the α+/γ2− interfaces (see Figure 1), benzodiazepine binding site ligands such as the class of pyrazoloquinolinones can bind at both these interfaces, eliciting different functional effects [19]. While still being in an early stage, the α+/β− interfaces are considered to be highly promising targets for novel therapeutic principles as well as a wide range of tool compounds to be utilized in (human brain) imaging or research applications [20].

Some benzodiazepines also bind at non-homologous sites located in the TMD [21,22]. These sites are thought to be the main site of action of intravenous anesthetics such as etomidate or propofol. Promiscuous binding of chemotypes that bind at the high-affinity benzodiazepine binding site at both extracellular and TMD sites has been observed unexpectedly often [22]. In this review, ligands of the high-affinity benzodiazepine sites, the extracellular α+/β− interfaces, and the TMD sites with which barbiturates and etomidate interact are covered.

### 1.3. Allosteric Ligands and Their Action on GABA_A_ Receptors

Ligands that bind to allosteric sites can have a multitude of effects on the receptor complex in the presence or absence of orthosteric ligands. The structural basis of the GABA_A_ receptor pharmacology has been reviewed recently based on a surge of structural data of heteropentameric receptors with various ligands present in ortho- and allosteric sites [23].

Many compounds can interact with several binding sites that are contained within a receptor. The ones that bind at the location of the endogenous ligand are known as orthosteric ligands, whereas compounds that interact in other binding sites that are different from the orthosteric binding site are known as allosteric ligands.

#### 1.3.1. Orthosteric Ligands

Orthosteric ligands can be classified into agonists, antagonists, or inverse agonists, based on the response that their binding causes to the receptor (definitions were taken from NIH, NIC dictionary). Agonists cause the same effect as the endogenous ligand that normally binds to the receptor. Antagonists attenuate or block the effect of an agonist. Inverse agonists bind to the receptor and produce the opposite pharmacological effect that would be produced by the endogenous ligand [24]. 

**Figure 2:**
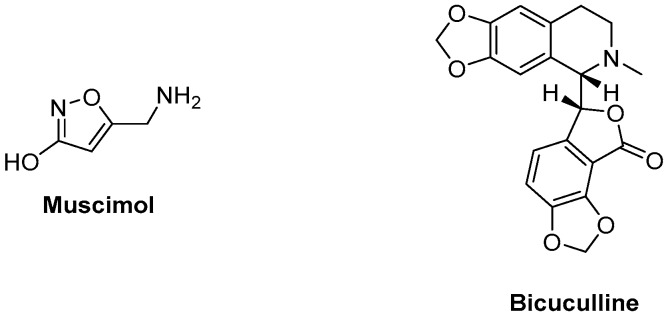
Examples of a GABA_A_ receptor agonist and antagonist.

An example of a GABA_A_ agonist is muscimol, whereas a well-known antagonist is bicuculline [25,26,27]. Figure 2 depicts the structure of examples of an agonist and an antagonist/inverse agonist of the GABA_A_ receptor. 

#### 1.3.2. Allosteric Ligands

When allosteric ligands interact with a receptor, they can either exert an effect in the receptor’s physiology or just bind but produce no modification on the receptor’s physiology. In the former case, some allosteric ligands can directly exert their effects without the orthosteric ligand present. On the other hand, allosteric ligands that require the presence of the endogenous ligand to exert an effect are commonly referred to as allosteric modulators.

**Figure 3:**
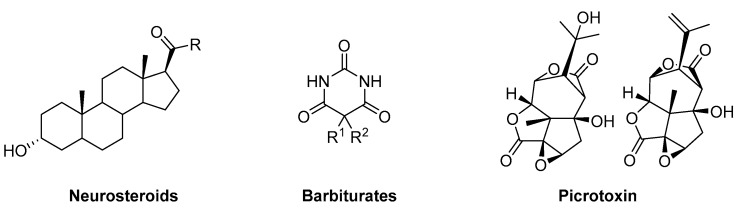
Examples of allosteric GABA_A_ receptor ligands.

Allosteric ligands that produce an enhanced response when interacting with the receptor in the absence of the endogenous ligand are known as allosteric functional agonists or termed GABAmimetics. Examples of these kinds of allosteric functional agonists are etomidate, barbiturates, or neuroactive steroids [28,29]. Similarly, allosteric ligands can reduce or block channel activity in non-competitive ways. An example of a GABA_A_ receptor blocker is picrotoxin, which has a binding site located within the channel pore [30]. Functional allosteric antagonism that is not based on a channel block can occur at a variety of allosteric sites, such as the effects elicited by inhibitory steroids [14]. Figure 3 depicts the structure of some examples of allosteric ligands, which impact the receptor function in the absence or presence of GABA.

Allosteric modulators (in the strict sense) require the presence of the endogenous ligand to exert their effects. In other words, they modulate the GABA-elicited channel response. In general, allosteric modulators are classified in the following way.

- Positive allosteric modulators (PAM): they enhance the effect of the endogenous ligand
- Negative allosteric modulators (NAM): they diminish the effect of the endogenous ligand
- Silent allosteric modulators (SAM): they do not influence the effects of the endogenous ligand, but they can compete with a PAM or a NAM for the occupation of a binding site [31].

**Figure 4:**
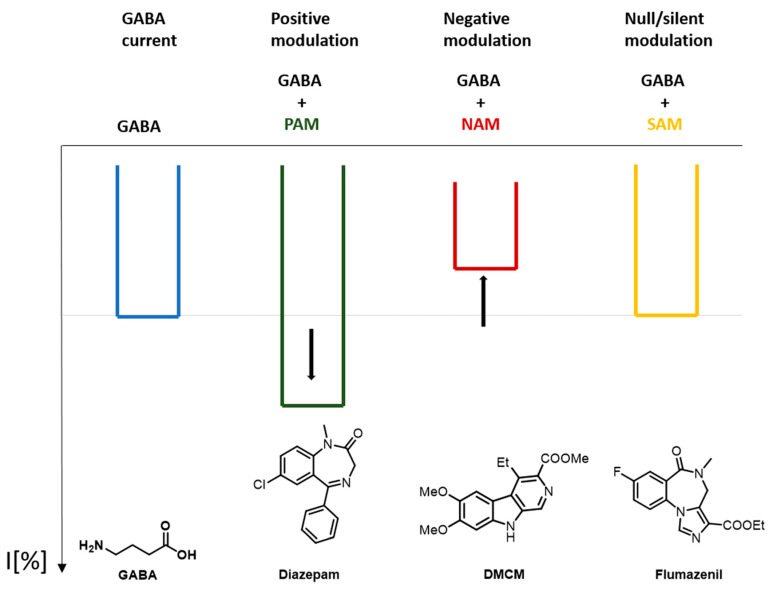
Effect on the response current caused by a GABA_A_ positive allosteric modulators (PAM), negative allosteric modulators (NAM), and silent allosteric modulators (SAM).

Figure 4 shows a schematic representation of the effect that a GABA_A_ receptor PAM, NAM, and SAM can produce in comparison to the response that GABA produces.

Since some ligands can fall into more than one category of these classifications, each receptor subtype must be considered separately for each case. Some ligands can act as a SAM in a certain receptor subtype, whereas, in other receptor subtypes, it can show a PAM behavior. Flumazenil is an example of a GABA_A_ receptor SAM [18]. Etomidate is another example of a GABA_A_ receptor ligand with a mixed behavior. Depending on the concentration that is being studied, etomidate can behave as an allosteric modulator or as an allosteric functional agonist.

The next chapter presents the most commonly used assays, which are used to characterize GABA_A_ receptor ligands. In terms of the specific activity of any ligand, it is important to note that terminology is very heterogeneous and has developed historically, and terms such as “modulator” and “agonist” are often used synonymously and without clear experimental evidence for one or the other mechanism of action.

## 2. Assays to Probe Interactions of Small Molecules with the Receptors, or with Specific Binding Sites

Historically, radioligands were used to test substances’ ability to displace or modulate a known ligand, where usually tritiated ligands are used and where brain membranes are used as a preparation of all receptors found in the brain. To test specific receptor subtypes, membrane preparations from cells used for recombinant expression are employed (typically HEK, COS, Ltk cells, etc.). The most commonly used radioligands interact with the orthosteric site, with the high-affinity benzodiazepine sites, and with the channel pore. 

Ligands of the orthosteric site (GABA sites) such as muscimol, GABA, gabazine (and likely many others) were and still are used. They display different degrees of specificity for different isoforms (subtypes). To indicate the challenges and limitations, in a recent study, it was shown that the majority of high-affinity muscimol sites in brain membranes are not the most expressed αβγ receptors, but are a smaller population of δ- containing receptors [32]. 

In this scenario, we are interested chiefly in allosteric ligands. Historically, radioligands for the high-affinity benzodiazepine sites predate the cloning of receptor subunits and were originally considered as ligands of an unidentified “benzodiazepine receptor.” Thus, ligands that displaced radiolabeled benzodiazepines were termed “agonists,” “antagonists,” and “inverse agonists” of the “benzodiazepine receptor.” This leads to the unfortunate situation that benzodiazepine site ligands are often referred to as “GABA_A_ receptor agonists or inverse agonists,” which is rather misleading. Several radioligands for high-affinity benzodiazepine sites exist and are widely used: flunitrazepam, flumazenil, Ro-15 4513 are the most commonly used ones. They do not show complete overlap in the subtypes to which they are binding with high affinity. Therefore, some radioligands bind to more receptor subtypes than others due to differential interactions with isoforms of α or γ subunits.

Radioligands that bind in a state-dependent way to the ion channel also exist, where a prominent example is EBOB. EBOB binding is state-dependent. Therefore, its binding is dependent on the absence or presence of GABA and often even responds to allosteric modulators. Thus, modulation (rather than displacement) of EBOB binding can also be used to detect ligands that bind at sites other than the channel lumen, but these sites then remain unspecified [33]. 

Limitations of radioligand based assays include the following. Competitive displacement is the gold standard to confirm binding site usage. Radioligand modulation helps to detect ligands that use unknown sites [33], but may fail to respond to sites that cause only small or no conformational changes (silent binders that may not be silent in the presence of other agents). 

As discussed in the previous chapter, ligands not only bind to the receptors, but they exert complex effects such as negative or positive modulation, or direct channel block, or allosteric “activation” on them. These effects can only be detected with functional assays. This more comprehensive approach directly assesses the effects of ligands on channel activity, or on agonist-elicited channel activity. The biggest disadvantage of functional studies is that the binding sites cannot be identified by the assay. Competitive functional assays are exceedingly challenging and often allosteric and competitive effects cannot be reliably distinguished. 

The most commonly used methods to determine ligand function comprise low and high throughput electrophysiological assays and diverse methods that are based on fluorescent probes that are charge-sensitive or, otherwise, coupled to changes in membrane potential. Functional assays are performed in recombinantly expressing cell systems to determine the effects of ligands on defined receptor species or on known mixtures of subunits that are expressed, or on ex vivo preparations such as acute brain slices, synaptosome or membrane preparations, or on primary cultured cells (typically hippocampal neurons). It has to be noted that the large diversity of receptor subtypes renders every assay incomplete in the sense that effects only on those receptors that are present in a given preparation can be captured. 

Due to the big complexity of GABA_A_ receptor-mediated pharmacology and the wide diversity of assays, which are used to investigate ligand properties such as affinity, potency, electrophysiological efficacy, and in vivo effects and efficacy, it is challenging to compare ligand characteristics across different studies. Even though, by now, it is well acknowledged that radioligand-based competition assays can fail to detect modulatory interactions, there is still a lack of consensus concerning functional testing. The large number of receptor subunits, the lacking knowledge about the precise number of existing subtypes, and the observation that neurons, glia cells, and non- neuronal cells express a broad and heterogeneous mixture of subunits documents that no single in vitro or ex vivo protocol can be designed and research relies on combining multiple assay types to define ligand effects on molecular species. A recent review illustrates the complexity and discusses many of the problems that are faced on the path toward receptor subtype-specific ligand characterization [16].

Generally, after binding or functional effects are confirmed in either heterologously expressed protein or suitable ex vivo preparations (such as brain membranes, acute brain slices, or neuronal cell cultures), a broad range of toxicological and behavioral testing will follow for the most promising compounds. The behavioral animal experiments are out of the scope of this review. As a very broad rule, compounds that are chiefly GABAmimetic or positive allosteric modulators display sedative-like and anticonvulsant-like effects while ortho- and allosteric antagonists and blockers will be anxiogenic and pro-convulsant. If individual subtypes respond differently, very complex combinations of effects can occur and raise hopes for the development of totally novel therapeutics that combine silent, PAM, and NAM characteristics toward different receptor subtypes [34]. 

## 3. Clinical Pharmacology of Allosteric Ligands Used in Human Medicine

Three large groups of heavily used GABAergics are allosteric modulators of GABA_A_ receptors, and additional indication groups exist and are summarized in this case as a fourth heterogeneous group.

### 3.1. Sedative General Anesthetics

**Figure 5:**
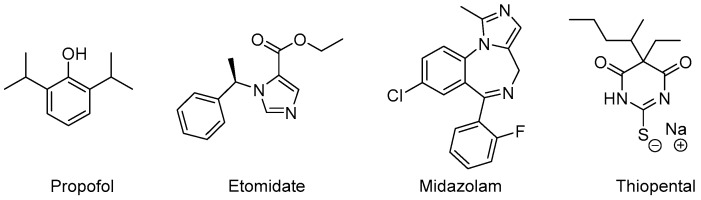
Anesthetic drugs with different allosteric mechanisms of action.

Drugs with reliable sedative-hypnotic, amnestic, anxiolytic, antinociceptive, and immobilizing properties represent an indispensable component of state-of-the-art general anesthesia. After the introduction of diethyl ether as the first viable anesthetic for surgery in the middle of the 19th century, drug discovery became a prime focus of research in the field of anesthesia [35]. The ongoing quest for novel compounds with rapid-onset, potent sedative-hypnotic effects, predictable clearance to inactive metabolites and minimal side effects has yielded a range of clinically established drugs. The vast majority of anesthetic compounds in use today interact with GABA_A_ receptors [36]. In this scenario, we provide a glance at the clinical roles of four important anesthetics, with the primary effects to rely predominantly on positive allosteric modulation of GABA_A_ receptors: Propofol, Etomidate, Midazolam, and Thiopental (Figure 5).

Propofol is a hydrophobic compound for intravenous application in a lipid-solution. It was first approved for use in the 1980s and has quickly advanced to become the most frequently administered intravenous drug for the induction of general anesthesia and a common choice for the maintenance of general anesthesia as well as procedural sedation [37]. Rapid inductions, rapid terminal half-life time, low incidence of postoperative nausea and vomiting, as well as rapid psychomotor recovery make it a very versatile hypnotic drug [38].

Propofol substantially inhibits sympathetic nerve activity, contributing to a significant, dose-dependent decrease of mean blood pressure [39] as well as moderate respiratory depression [40]. While these cardiopulmonary side-effects are predictable and well manageable in most settings, they often present a challenge in hemodynamically constrained patient populations. Other adverse events include pain on the injection site and less commonly hyperlipidemia resulting from lipid formulation.

Etomidate is an imidazole derivative, where its anesthetic activity was coincidentally discovered during animal testing as an antifungal agent in the 1960s. Its rapid onset of effects can be explained by the compound’s lipid solubility and large non-ionized fraction at physiological pH and make it a common choice for induction of general anesthesia and short procedures requiring deep sedation. The prolonged application of etomidate, however, is not feasible due to the incidence of adrenocortical suppression, which negatively impacts the outcome, especially in critically-ill populations. Compared to propofol, etomidate only causes a modest decrease of arterial blood pressure and leaves the respiratory system largely unaffected [41].

Midazolam is a short-acting, rapid-onset benzodiazepine primarily used during procedural sedation. Its low hemodynamic side-effects also make it a common choice for general anesthesia during cardiac surgery. Its amnestic effects are advantageous in treating intraoperative awareness [42] and its application may be correlated with a decreased likelihood of delayed recall of procedural sedation [43]. As with other benzodiazepines, paradoxical excitement, cognitive impairment, and prolonged recovery time present known complications.

Thiopental is an ultra-short acting barbiturate derivative. Barbiturates were used extensively over several decades to induce and maintain general anesthesia. They have been largely replaced by newer compounds with safer pharmacokinetic and pharmacodynamic profiles. Thiopental remains the only anesthetically used barbiturate with a firm place in today’s surgery rooms and emergency departments.

Many additional anesthetic compounds such as inhalational fluranes and ketamine also allosterically modulate GABA_A_ receptors but rely on other primary mechanisms of action.

### 3.2. Prescription Hypnotics and Sleeping Aids

GABA_A_ receptors present the prime molecular targets in the pharmacological treatment of insomnia. Barbiturates undoubtedly played a historically significant role as prescription hypnotics but have been largely replaced by safer options over the years. Their low therapeutic index paired with high tolerance formation and addiction potential contribute to the significant risks associated with their use [44].

Benzodiazepines are known for their ability to promote sleep, dampen anxiety, and provide muscle relaxation with a much broader therapeutic index than barbiturates. Their aggressive marketing and extensive prescription during recent decades after their market introduction are now being increasingly criticized after tremendous evidence for risks associated with long-term usage emerged. Prescription of benzodiazepines such as temazepam and nitrazepam still constitute a significant fraction of prescribed hypnotics [45]. The largest fraction of prescribed treatments for insomnia today is newer non-benzodiazepines such as zolpidem and zopiclone (Z-Drugs). While they are promoted and attributed to possessing greater benefits and fewer side effects than benzodiazepines, there is no evidence of significant differences in clinical effectiveness and safety [46].

Many nature-derived compounds of which some are used as OTC sleep-aids, also seem to mediate their sleep-promoting effects via interactions with GABA_A_ receptors [47].

The development of new drugs with high efficacy, lower addiction liability, and lower incidence of paradoxical reactions remains imperative in order to create safer long-term treatment options for insomnia.

### 3.3. Anticonvulsants

Several different antiepileptic drugs with various known and unknown mechanisms of action are in use for long-term prophylactic treatment. The treatment of acute seizures and status epilepticus, however, requires potent and fast-acting drugs capable of effectively disrupting the convulsive state. Many of these compounds are modulators of GABA_A_ receptors. Parenteral benzodiazepines such as midazolam, lorazepam, and diazepam are recommended during the initial phase of therapy, which begins after 5 min of a persistent seizure. If the seizure continues after a duration of 20 min, a second treatment phase begins, which consists of, not predominantly, GABAergic drugs such as fosphenytoin, valproic acid, or levetiracetam. Due to a higher incidence of adverse events, phenobarbital is only recommended in case the other options are unavailable. At the 40-min mark, a third treatment phase begins, where anesthetic doses of thiopental, midazolam, or propofol should be considered [48].

### 3.4. Additional Indication Groups for Therapeutically Used Allosteric Modulators of GABA_A_ Receptors Exist

Other indication groups for benzodiazepines include anxiolysis and central muscle relaxation. In such cases, sedative effects are unwanted and tolerance formation limits the duration of effective treatment. Since there are differences in the effect profiles of benzodiazepines, specific derivatives are recommended for specific indications, which are not only based on their pharmacokinetic profiles [45]. 

Barbiturates are still regularly used in the management of severe traumatic brain injury, where lowering intracranial pressure and optimization of cerebral oxygenation are critical [49].

### 3.5. PET and SPECT Imaging Ligands

Allosteric ligands of specific groups of GABA_A_ receptor subtypes are not only in use as therapeutics but also as isotope-labeled imaging ligands for positron emission tomography (PET) or single-photon computed tomography (SPECT) to investigate altered receptor expression patterns in a wide range of neuropsychiatric disorders [50]. At this time, all commonly used ligands for human brain imaging are ligands of some of the high-affinity benzodiazepine binding sites. Prominent examples are flumazenil, iomazenil, and Ro 15-4513. Imaging ligands that interact with other receptor subtypes and do not rely on the presence of a γ-subunit would be highly desired.

## 4. Allosteric Ligands and Their Synthetic Accessibility

Many heterocyclic scaffolds have been investigated as GABA_A_ receptor ligands. In addition, it turns out that many of them contain nitrogen heterocycles. In fact, a large part contains even fused heterocycles, which represent scaffolds that are often complex to synthesize. Within this chapter, the more recent (last 10 years) synthetic developments in the large field of GABA_A_ receptor ligands are summarized. This means that the selection of examples is based on new or improved synthetic methods that have been applied, and not on improved biological activity. Hence, examples synthesized via long-standing synthetic procedures are not included even though they might show interesting properties. For completion, the pharmacological data reported along with the synthesis are briefly summarized. Additionally, the subchapters are ordered according to the chemical relation, and not according to activity, a binding site, or factors alike.

The chemotypes herein covered are a non-limiting list of the GABA_A_ receptor modulators that have been, so far, discovered and synthesized via new or improved routes within the last 10 years. For this reason, synthetic accessibility of heterocycles is reviewed. 

### 4.1. Monocyclic GABA_A_ Receptor Modulators

#### 4.1.1. Monocyclic GABA_A_ Receptor Modulators with 5-Membered Rings

##### Imidazole Derivatives

**Figure 6:**
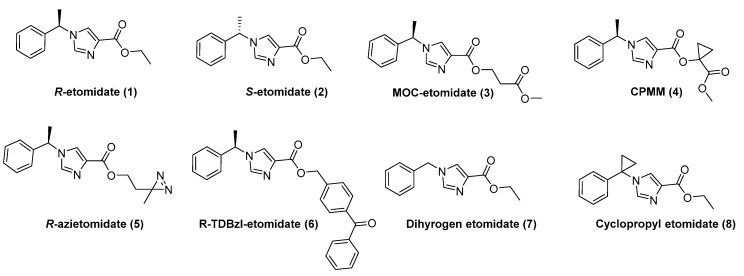
Structures of etomidate enantiomers and analogs.

Etomidate is a general anesthetic that not only positively allosterically modulates the GABA_A_ receptor, but also, at subclinical doses, it can activate the GABA_A_ receptor in the absence of GABA. β2 and or β3-subunit containing receptors are much more sensitive to etomidate than β1 containing receptors. Another relevant etomidate derivative is methoxycarbonyl metomidate (MOC-etomidate), which modulates GABA_A_ receptors with similar potency, but it is quickly metabolized and inactivated [51,52]. Azietomidate also enhances the GABA_A_ receptor function whereas TFD-etomidate has a low GABA_A_ receptor activity. Achiral etomidate analogs have been designed and tested, such as dihydrogen etomidate and cyclopropyl etomidate, but both of them have low potencies, comparable to *S*-etomidate [53]. The structures of these etomidate derivatives are depicted in Figure 6.

**Scheme 1:**
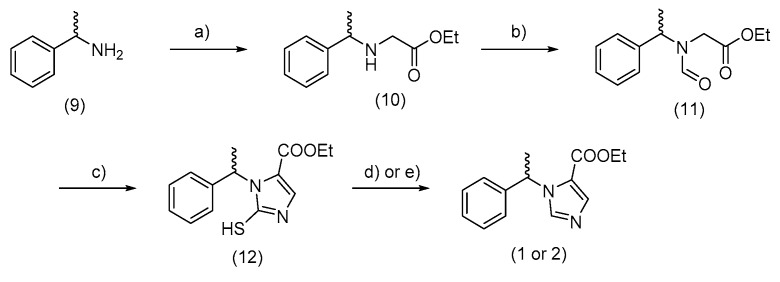
a) Ethyl chloroacetate, TEA, DMF, rt., overnight, yield not reported. b) HCOOH, xylene, reflux, few hours, quant. yield. c) 1. NaOEt/HCOOEt, EtOH, r.t., 2. KSCN/HCl. d) 1. HNO_3_/NaNO_2_, 25–35 °C, 2 h, r.t. 2. Na_2_CO_3_, yield not reported. e) 30% H_2_O_2_, Toluene, H_2_O, 40–45 °C, yield not reported.

The synthesis of etomidate was reported for the first time in the US patent 3,354,173. In this process, methyl benzylamine (compound **9**) is treated with ethyl chloroacetate to yield *N*-ethoxycarbonylmethyl-*N*-1-phenylethylamine (compound **10**). This product is subsequently formylated with formic acid and the resulting *N*-ethoxycarbonylmethyl-*N*-formyl-*N*-l-phenylethylamine is again C-formylated with ethyl formate and sodium ethoxide, which yields compound **11** as a result. The product (compound **11**) is treated with thiocyanate in hydrochloric acid. This causes the *N*-formamide protecting group to hydrolize and the resulting product with an aldehyde moiety will undergo a Marckwald heterocyclization, which forms compound **12**. In the final step, the thiol group is removed under oxidative conditions with nitric and nitrous acids and further treated with IPA HCl to achieve the corresponding hydrochloride salt of the etomidate. Neutralization yielded etomidate (compound **1** or **2**) as a free base (Scheme 1). [54,55].

Subsequently, a slightly modified approach was described in which the last step is performed by using hydrogen peroxide as an oxidizing agent to perform the final desulfurization in order to avoid highly exothermic conditions and the generation of nitrous oxide gas. This modified approach is only reported with the *R*-enantiomer of etomidate (compound **1**), whereas the original procedure was performed with the corresponding racemate (Scheme 1) [54].

**Scheme 2:**
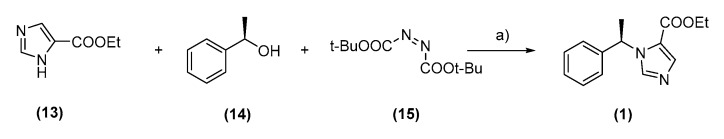
a) 1. PPh_3_, THF, −40 °C, 70%.

An alternative approach was reported by using a Mitsunobu alkylation of ethyl imidazole carboxylate (compound **13**), which was followed by a palladium-catalyzed cyclization in very good yields. Racemization was avoided by performing the reaction at low temperatures (Scheme 2) [56].

**Scheme 3:**
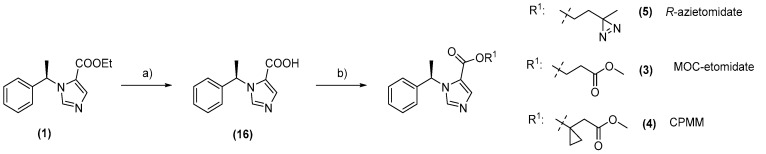
a) NaOH 10 M, 100 °C, 1 h. b) R^1^-OH, 4-(dimethylamino)pyridine, dicyclohexyl-carbodiimide, or 1-ethyl-3-[3(dimethylamino)propyl]carbodiimide hydrochloride.

The synthesis of etomidate derivatives with a space linker (MOC-etomidate, (compound **3**), cyclopropyl etomidate (compound **4**), and azietomidate (compound **5**)) can be achieved from the desired etomidate enantiomer (compound **1** or **2**) in a two-step procedure (Scheme 3). Azietomidate (compound **15**) can be easily obtained by treating etomidate in its chirally pure desired form (compound **1** or **2**) with an excess of a base in order to hydrolyze the ester moiety. The resulting carboxylic acid was re-esterified with 3-azibutanol in the presence of dicyclohexylcarbodiimide (DCC) and a nucleophilic catalyst, 4-(dimethylamino)pyridine (DMAP), to yield azietomidate (compound **5**) as a product [57]. This same approach is also reported to obtain the MOC-Etomidate (compound **3**) and cyclopropyl methoxycarbonyl metomidate (compound **4**), but using the corresponding alcohol instead [58].

**Scheme 4:**
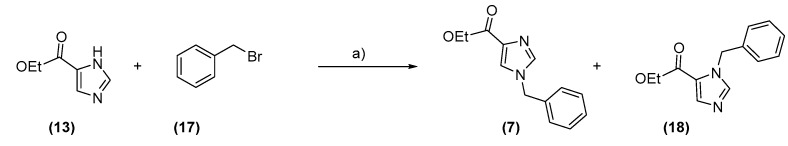
a) tetra-*n*-butyl-ammonium bromide, NaOH aq 20%, toluene, yield not reported.

The dihydrogen derivative of etomidate (compound **7**) was synthesized with a phase transfer–catalyzed *N*-alkylation of imidazole ethyl ester (compound **13**) with benzyl bromide (compound **17**) and tetra-*n*-butylammonium bromide. This procedure yielded the desired dihydrogen etomidate as well as its respective isomer (compound **18**) (Scheme 4) [59].

##### Arylalkylazoles

**Figure 7:**
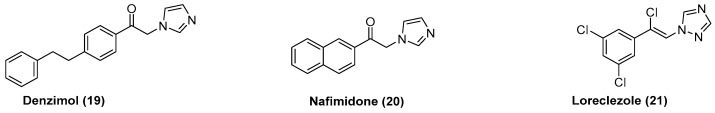
Arylalkylazoles.

Compounds containing an azolic ring have been reported as ligands for the GABA_A_ receptors. Among them, compounds such as loreclezole (compound **21**), nafimidone (compound **20**), and denzimol (compound **19**) belong to the class of arylalkylazoles, which is a class of experimental anticonvulsants (Figure 7).

**Scheme 5:**
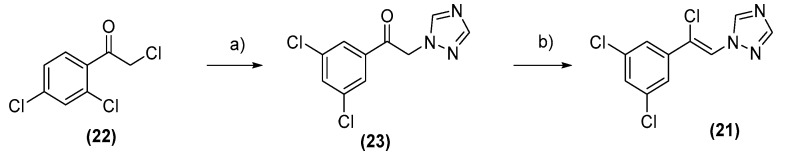
a) K_2_CO_3_, 1*H*-1,2,4-triazole, CH_3_CN, MW 85 °C (P 50 W), 50 min, 98%. b) 1,2-dichloroethane, PCl_5_, reflux, 1.3 h.

Loreclezole (compound **21**) was shown to be anticonvulsant in animals, and features β-isoform selectivity [60]. In particular, selectivity towards β2 and β3 over β1 containing receptors has been demonstrated with mutational studies and points to an overlapping site of action with etomidate [61]. Loreclezole (compound **21**) is synthesized in a two-step synthesis, as outlined in Scheme 5. The first step, an *N*^1^-alkylation of the 1*H*-1,2,4-triazole with compound **22** is performed under microwave irradiation to yield compound **23** [62,63]. In the second step, 1-(3,5-dichlorophenyl)-2-(1*H*-1,2,4-triazol-1-yl)ethan-1-one (compound **23**) is transformed to loreclezole (compound **21**) upon treatment with phosphorus pentachloride.

**Scheme 6:**
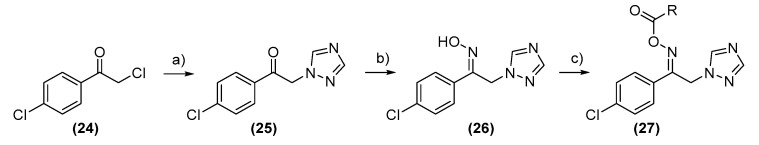
a) 1,2,4-triazole, Na, CH_3_OH, reflux. b) NH_2_OH·HCl, C_2_H_5_OH, pH 14, reflux. c) R-COOH, DCC, DMAP, DCM, ethereal g HCl.

Modifications of the loreclezole scaffold have been investigated to discover new potential anticonvulsants. The synthesis of these derivatives is shown in Scheme 6. After alkylation of 1,2,4-triazole with 2,4′-dichloroacetophenone (compound **24**). The hydroxylamine 26 was obtained by refluxing with hydroxylamine hydrochloride under basic conditions. Lastly, esterification using DCC as a coupling agent yielded the final compound 27.

**Scheme 7:**
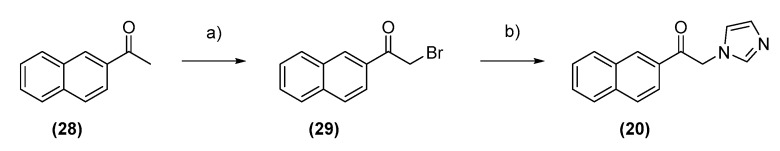
a) Br_2,_ glacial acetic acid, ice, overnight 80%. b) 1,2,4-triazole, DMF, 0 °C, 2 h, 45%.

Similarly, nafimidone (compound **20**) is synthesized, as shown in Scheme 7 [64,65,66], and also denzimol (compound **19**) is synthesized in a similar fashion, starting from the corresponding α-haloalkyl ketones [66].

#### 4.1.2. Monocyclic GABA_A_ Receptor Modulators with Six-Membered Rings

##### Barbiturates

Barbiturates (structures **30** and **31**) are derivatives of barbituric acid, which can positively modulate the GABA_A_ receptor and, at higher concentrations, can activate the receptor without the endogenous ligand [67,68]. 

The synthesis of this family of compounds has been reviewed by Vardanyan and Hruby [67]. Thus, only contributions since then are discussed in this chapter. Traditionally, barbiturates can be prepared by a condensation reaction of urea and the corresponding malonic ester with sodium alkoxide in ethanol or DMSO [2,67]. A disadvantage of this traditional procedure is that it yields barbiturate derivatives in a modest yield due to several well-known side reactions. Additionally, this procedure requires dry solvents, high temperatures, inert atmospheres, and metallic sodium, which limits the scope of the derivatives that can be obtained [2]. 

**Scheme 8:**
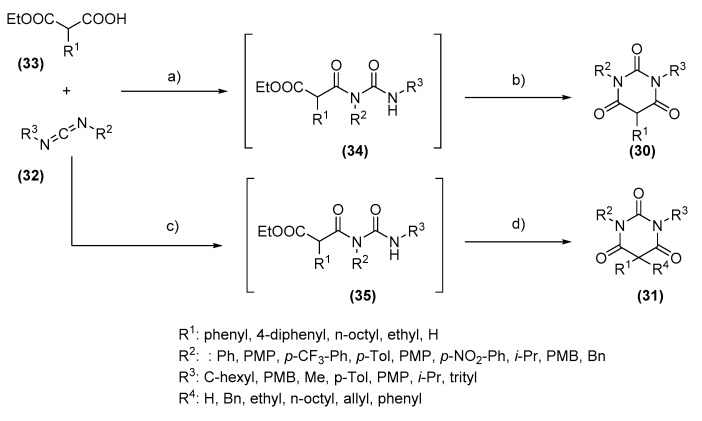
a) Solvent, r.t., 12 h, b) 2 N NaOH, H_3_O^+^, solvent, c) ACN, r.t., overnight, sealed tube, and d) K_2_CO_3_, R^4^-X, ACN, 120 °C, sealed tube.

Volonterio and Zanda developed a one-pot procedure to synthesize 1,3,5-substituted and 1,3,5,5-substituted barbiturates (Scheme 8). In this procedure, carbodiimides (structure **32**) reacted with malonic acid monoethyl esters (structure 33) in order to yield *N*-acylurea intermediates (structure **34**) that would be, afterward, cyclized by adding a base and the desired electrophile (structure **30**). Besides being a single-step strategy, the advantage of this procedure is that all reactions were performed under mild conditions. The solvents employed were THF, dioxane, acetonitrile, or DCM, even though dioxane provided, in general, the best results. With this protocol, the yields mostly were superior to 60% [2].

A limiting step of this procedure was identified for dialkyl carbodiimides that were employed as reagents (i.e., diisopropyl carbodiimide). In these cases, decarboxylation of the malonate (structure 33) occurred. Thus, reactions with this type of carbodiimides were performed in a less polar solvent (DCM) and in the presence of TMP in order to enhance the O-N acyl migration process. This modification satisfactorily resolved the issue and yielded respective desired products in yields over 67% [2]. 

C-monosubstituted barbiturates (structure **30**) were obtained by quenching the barbituric carbanion with protic acids and using 2N NaOH as a base. However, in the case of the tetrasubstituted barbiturates, it was found that some carbanions of barbiturates failed to react with their respective electrophiles. Thus, a slightly modified procedure was developed, where anhydrous K_2_CO_3_ was employed as a base. ACN became the solvent of choice and the reaction was carried out at 120 °C in a sealed tube. This protocol yielded the desired tetrasubstituted barbiturates (structure **31**) in yields superior to 65% (Scheme 8). It is noteworthy to mention that this alkylation protocol was also directly tested with barbituric acid as a starting point with satisfactory yields (Scheme not shown) [2]. 

**Scheme 9:**
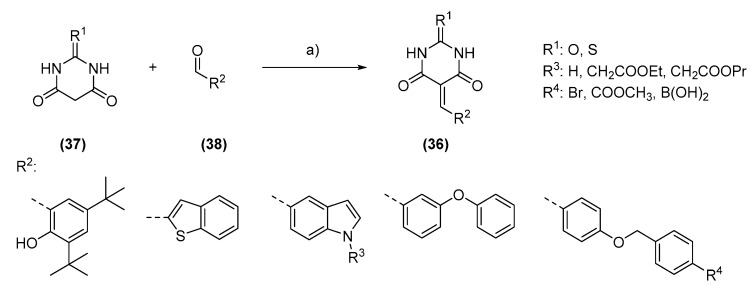
a) CH_3_COOH or CH_3_COONa, reflux, 12–36 h.

Additionally, another synthesis of heterocyclic-substituted barbiturates (structure **36**) was reported by performing a Knoevenagel condensation of barbituric acid and/or thiobarbituric acid (structure **37**) and differently substituted aldehydes (structure **38**). This procedure was performed either in the presence of acetic acid or in the presence of sodium acetate. Modified conditions were also performed under microwave irradiation, which substantially improved yields. This procedure enabled the access to barbituric acid derivatives (structure **36**) with different aromatic or heteroaromatic substituents and, thus, represents a methodology that enhances the diversity of this scaffold (Scheme 9) [69].

### 4.2. Fused Bicyclic GABA_A_ Receptor Modulators

#### 4.2.1. Fused Bicyclic GABA_A_ Receptor Modulators Containing One Nitrogen

##### Quinolones

**Scheme 10:**
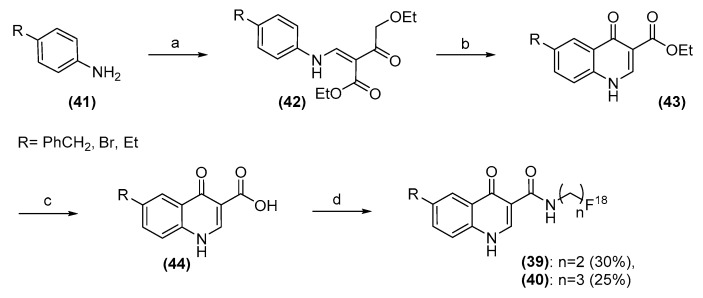
a) diethyl ethoxymethylenemalonate, 3 h, 120 °C, 80%. (b) diphenyl ether, 1 h, reflux, 50%. (c) NaOH (2 N), ethanol, 2 h, reflux, 90%. (d) 2-fluoroethylamine/3-fluoropropylamine, trifluoroacetate, HATU, DIPEA, DMF, 12 h, rt.

4-Quinolinone derivatives have been reported as high-affinity ligands at the BZD binding site of GABA_A_ receptors [70]. More recently, this scaffold was used for the synthesis of fluoro-labeled compounds (compounds **39** and **40**) as PET radiotracers for the GABA_A_ receptors [71]. Starting from **41**, the addition of diethyl ethoxymethylenemalonate yielded **42** that was heated in the presence of diphenyl ether to deliver **43**. Hydrolysis of the ester **43** and amide formation in the presence of HATU led to (compounds **39** and **40**) (Scheme 10). Among these, compound **45** (structure **39**, R=CH_2_Ph) was found to be a potent ligand of the GABA_A_ receptors (Ki = 0.70 ± 0.66 nM).

**Figure 8:**
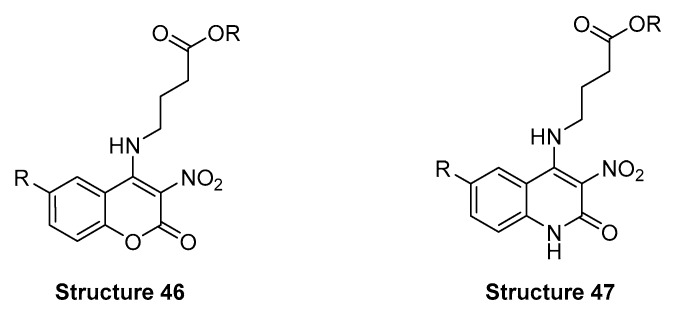
Coumarin derivatives.

**Scheme 11:**
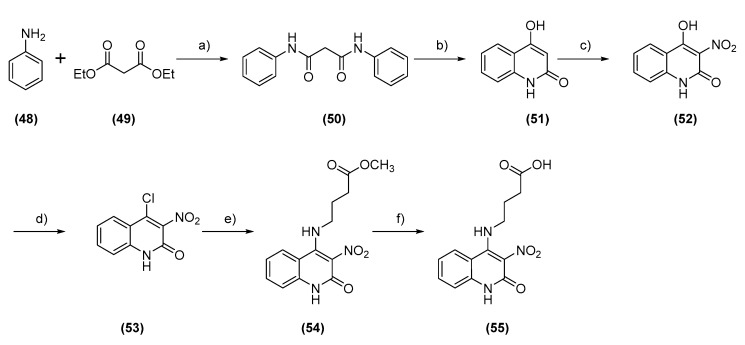
a) 210 °C, 5 h, 76%. b) Polyphosphoric acid, 150 °C, 5 h, 73. c) HNO_3_, 75 °C, 12 min, 93%. d) POCl_3_, TBBAB, MeCN, 40 °C, 30 min, reflux, 22 min, 60%. e) Py, reflux, 2 h, 63%. (f) NaOH, H_2_O, MeOH, reflux, 40 min, 64%.

Other quinolinone derivatives have been reported as anticonvulsants. Starting from the pharmacophore of nitrocoumarine-derivatives (structure 46, Figure 8), 4 amino-3-nitro-quinolin-2-one derivatives were designed (structure **47**, Figure 8) [72]. These structures are synthesized as displayed in Scheme 11 the reaction of malonic acid ester (compound **49**) and aniline (compound **48**) yielded compound **50**. Compound **51** was obtained via cyclo-condensation of compound **50** in the presence of polyphosphoric acid. In the following steps, nitration and chlorination were performed to deliver compound **53**. Lastly, the reaction with GABA methyl ester and the following hydrolysis delivered compound **55** (Scheme 11). Additionally, structure **47** showed anticonvulsant activity. In the same work, 4-amino-3-nitro-1-thiocoumarins were investigated. However, the thio-coumarin derivatives were found to have a lower anticonvulsant activity compared to the coumarin derivatives (O-containing).

##### Indol-3-yl glyoxyl Derivatives

**Figure 9:**
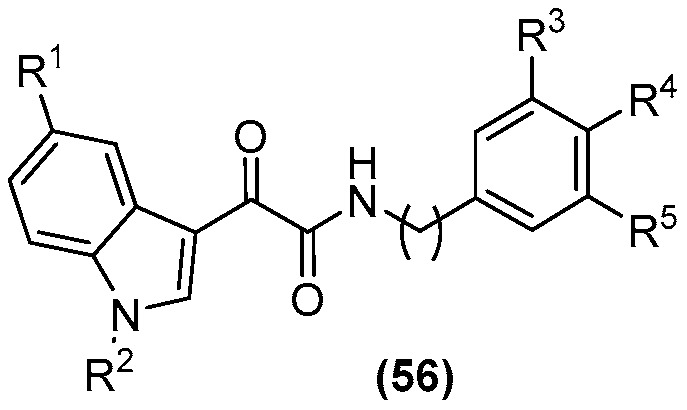
Structure of Indol-3-yl-glyoxyl scaffold.

The indol-3-yl glyoxyl scaffold (structure **56**) has also been explored as a template for designing GABA_A_ receptor ligands. Efficacy on co-application with GABA has been screened for several derivatives and some derivatives behaved as positive modulators with efficacies comparable to diazepam. The general structure of this scaffold is depicted in Figure 9.

**Scheme 12:**
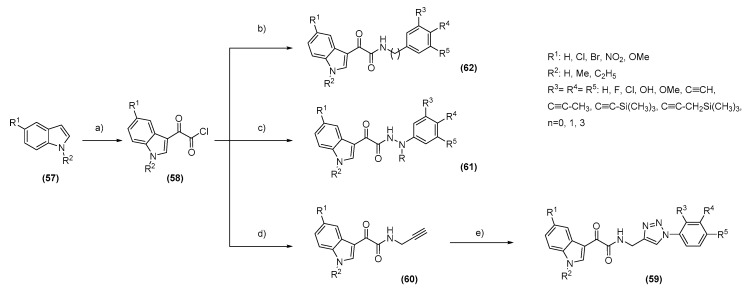
a) Oxalyl chloride, dry diethyl ether, r.t., 30 min, quant. b) NH-Aryl, TEA, Toluene, r.t. c) Arylhydrazine hydrochloride, TEA, toluene, r.t., 24–36 h. d) Propargyl amine, dry Pyridine, r.t., 8 h, quant. e) Aryl-azide, CuSO_4_·H_2_O, sodium ascorbate, *t*-BuOH/H_2_O (1:1), r.t., 8 h.

Synthesis of this scaffold (structure **56**) is reported by first performing an acylation of the accordingly substituted indole (structure **57**) with oxalyl chloride in anhydrous ether [73] and, afterward, treating the resulting compound with the desired glyoxalyl chloride (structure **58**) with either the corresponding hydrazine hydrochloride (structure **61**) in the presence of a base [74] or the corresponding amine (structure **62**) [75]. Alternatively, derivatives with a heterocyclic triazole moiety (structure **59**) can be prepared by reacting the corresponding indole-3-glyoxalyl chloride (structure **58**) with propargyl amine to afford the corresponding derivative with a terminal alkyne (structure **60**). The alkyne moiety was cyclized with click chemistry conditions using the accordingly substituted azide (Scheme 12) [76].

#### 4.2.2. Fused Bicyclic GABA_A_ Receptor Modulators Containing Two Nitrogens and 5–6 Membered Rings

##### Benzimidazoles

**Figure 10:**
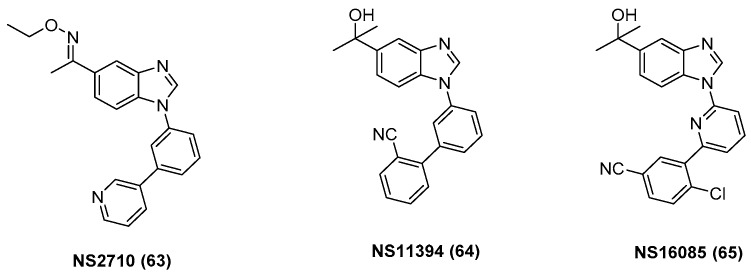
Structure of benzimidazole derivatives.

Benzimidazoles are a scaffold that was first reported in 1999 as modulators of the GABA_A_ receptor with the aim to develop functionally selective ligands [77]. Figure 10 depicts examples of this family of compounds are NS 2710 (compound **63**), NS 11,394 (compound **64**), and NS 16,085 (compound **65**). NS 11,394 (compound **64**) is a positive allosteric modulator for which functional selectivity has been explored in a small panel of α5-, α3-, α2-, and α1-containing GABA_A_ receptors. [78,79]. The profiles of NS 2710 (compound **63**) and NS16085 (compound **65**) showed subtle differences in comparison [79,80]. 

**Scheme 13:**
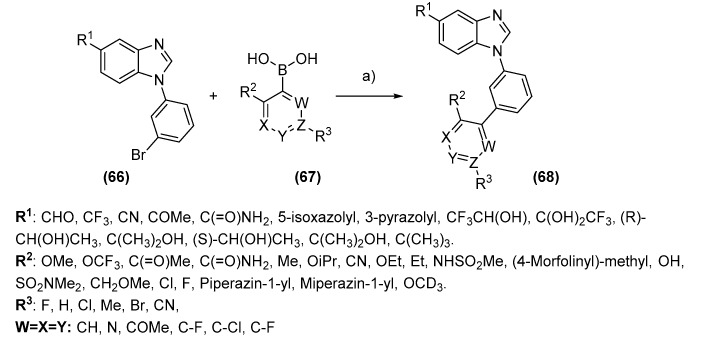
a) (Ph_3_P)_2_PdCl_2_, Na_2_CO_3_, DME/H_2_O.

Synthetic accessibility of this scaffold is reported in general via a Suzuki-Miyaura coupling, using 3-bromophenyl-1*H*-benzo[*d*]imidazole with the desired substituent in position 5, the accordingly substituted aryl boronic acid, sodium carbonate as a base, and 5 mol% of the catalyst (Scheme 13) [81]. This procedure is compatible with several functional groups in position 5 (alkyl alcohols, trifluoromethyl, ketones, aldehydes, nitriles, amides, and heterocyclic rings such as oxazoles, pyrazoles, and imidazoles). Additionally, the corresponding arylboronic acids can be substituted with halogens, methoxy groups, short-chain alkyl groups such as methyl or ethyl, sulfonamides, or even a heterocyclic ring such as a pyridine.

**Scheme 14:**
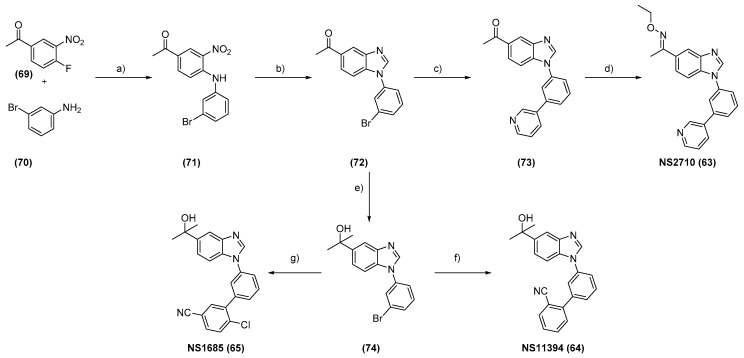
a) NMP, 80 °C, N_2_ atm, 72 h, 95% yield. b) Fe, formic acid, acetic acid, EtOAc, 70 °C, 30 h, 88% yield. c) Suzuki coupling, conditions not specified, yield not reported. d) *o*-ethylene hydroxylamine HCl, EtOH abs, reflux, 1.5 h, 60% e) MeMgCl, THF, rt, overnight, 57% f) 2-cyanophenyl boronic acid, sodium carbonate, dichlorobis(triphenylphosphine)palladium, 1,2-Dimethoyxethane, 90 °C, 6–8 h, yield not reported. g) (2-Chloro-5-cyanophenyl)boronic acid, sodium carbonate, dichlorobis(triphenylphosphine)palladium, 1,2-Dimethoyxethane, 90 °C, 6–8 h, yield not reported [81,82,83].

Compounds such as NS11394 (compound **64)** and NS16085 (compound **65**) can be obtained using intermediate **72** as a starting point (Scheme 14). The synthesis of **72** begins with an SNAr of 4-fluoro-3-nitro-acetophenone (compound **69**) with 3-bromoaniline (compound **70**) giving compound **71**. Compound **71** is converted to the key intermediate **72** via reduction of the nitro group to the corresponding ortho-dianiline and cyclization to the benzimidazole using formic acid as a methylene source in a one-step fashion. In order to obtain compound NS 2710 (compound **63**), intermediate **72** undergoes a Suzuki-Miyaura coupling using pyridine-4-ylboronic acid and, subsequently, the resulting product (compound **73**) is treated with *o*-ethylhydroxylamine hydrochloride to obtain the corresponding oxime (compound **63**). Further treatment of intermediate **72** with methylmagnesium chloride yielded the tertiary alcohol **74**. The synthesis of both NS11394 (compound **64**) and NS1685 (compound **65**) requires the Suzuki-Miyaura coupling of intermediate **74** with the corresponding aryl boronic acid.

##### Imidazopyridines

Zolpidem (compound **75**) (Ambien ^®^) is the most prominent representative of the imidazopyridine compound class. It belongs to the “Z-Drugs,” which take their name from the initial letter of the drugs name such as zolpidem (Ambien ^®^), zaleplon (Sonata^®^), and zopiclone (Imovane, Zimovane, Dopareel^®^). [84] Z-drugs are non-benzodiazepines that act as positive modulators at some of the same binding sites as the benzodiazepines, but with a different selectivity profile toward the α-isoforms. They are mostly prescribed in treating insomnia and sleeping disorders [85]. Zolpidem has a short elimination half-life and a rapid onset of effect, which makes it a short-acting sleeping aid [86,87].

**Scheme 15:**
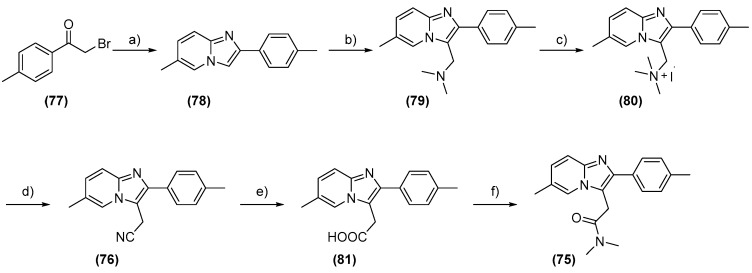
a) 2-Amino-5-methylpyridine, NaHCO_3_, EtOH, ∆. (b) Acetic acid, aq. dimethylamine, formalin, rt (c) Acetone, CH_3_I, ∆ (d) NaCN, EtOH, ∆ (e) KOH, EtOH/∆ (f) Carbonyldiimidazole, THF, dimethylamine, rt.

Different strategies have been investigated for the synthesis of Zolpidem [88]. The most common routes involve the synthesis of the acetonitrile intermediate **76** [89,90,91]: treatment of 2-bromo-1-(*p*-tolyl)ethan-1-one (compound **77**) with 2-amino-5-methylpyridine yielded compound **78**. The addition of formalin, dimethylamine, and acetic acid lead to the dimethylamino-imidazopyridine (compound **70**), which, upon addition of methyl iodide, followed by the addition of sodium cyanate delivers intermediate **76**. In the last two steps, hydrolysis and amidation are performed to obtain zolpidem (compound **75**), as depicted in Scheme 15.

**Scheme 16:**
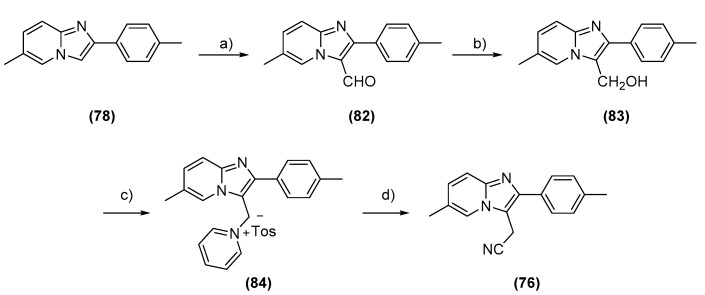
a) Oxalyl chloride, DMF −30 °C to 0 °C, 30 min, yield not reported. b) NaBH_4,_ rt, 8 h, yield not reported. c) *p*-toluenesulfonyl chloride, pyridine, rt, 8 h, yield not reported. d) NaCN, H_2_O, reflux, 2-5 h, yield not reported.

Other strategies involve a formylation approach for the synthesis of the acetonitrile intermediate **76** (Scheme 16) [91]. Formylation is obtained by the treatment of **78** with oxalyl chloride and dimethylformamide, which is followed by a reduction to yield alcohol **83**. This is, subsequently, transformed to the pyridinium tosylate salt **84**. The addition of sodium cyanide to **84** leads to the formation of **76**. However, both procedures are not suitable for large-scale production due to the toxicity of the reagents (oxalyl chloride, methyl iodide), their moisture sensitivity (carbonyldiimidazole), their volatility (methyl iodide), and unsatisfactory yields. Therefore, different strategies have been investigated. As reported by Eswaraiah et al. [92], intermediate **76** can be obtained using ethyl chloroformate for the synthesis of the quaternary salt that, upon treatment with sodium cyanide, delivered the key intermediate **76** (scheme not shown). Furthermore, the treatment of acid **81** with pivaloyl chloride in triethylamine followed by the addition of aqueous dimethylamine solution led to the formation of zolpidem in good yields.

**Scheme 17:**
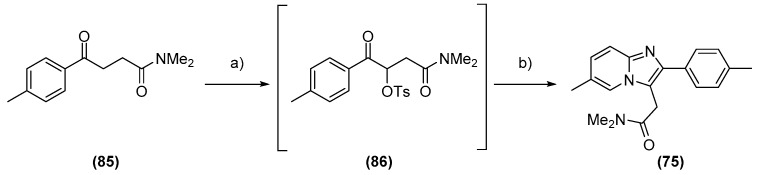
a) Iodosobenzene (PhIO), *p*-TsOH/CH_3_CN, reflux 2 h. b) 2-Amino-5-methyl pyridine, reflux 2 h, 47%.

Another reported strategy involves the condensation of the α-tosyloxy ketone **86**, obtained from *N,N*-dimethyl-4-oxo-4-tolylbutanamide **85** upon treatment with HTIB, with 2-amino-5-methylpyridine (Scheme 17) [88].

**Scheme 18:**
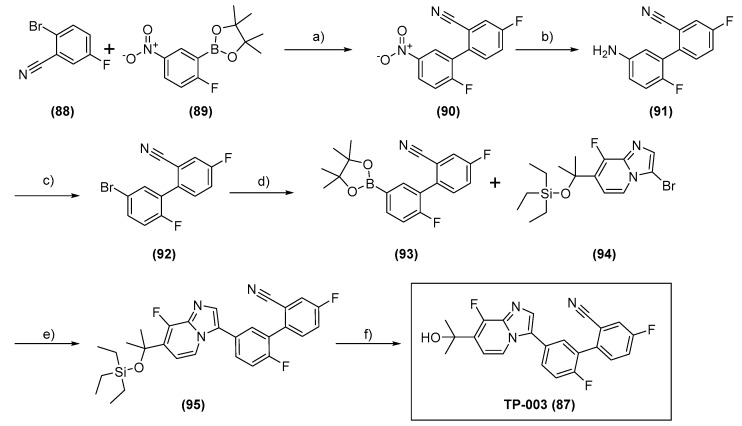
a) KOAc, Pd(DPPF)Cl_2_, 2% DMSO in dioxane, 90 °C, 14 h, 100%. b) 1. SnCl_2_·2H_2_O, EtOH:THF (1:1), rt, 12 h; 2. 2 N NaOH, rt, 1 h, 75%. c) 1. HBr (aq. 48%), NaNO_2_, 0 °C, 2 h, 2. CuBr, NH_4_OH 0 °C to 50 °C, 10 min, 64%. d) KOAc, bis(pinacolato)diboron, PdCl_2_[P(cy)_3_], 1% DMSO in dioxane, 90 °C, 16 h, 100%. e) Pd(PPh_3_)_4_ (5 mol%), 2 M Na_2_CO_3_, THF, 75 °C, 16 h, 60%. f) HCl (aq.37%), EtOH, 95%.

Other structures containing the imidazopyridine scaffold, such as TP-003 (compound **87**) are described as GABA_A_ modulators. This chemotype reflects an effort to achieve anxio-selective activity (with reduced sedation) by functional selectivity toward receptor subtypes containing specific α-isoforms. TP-003 (compound **87**) was initially proposed as an α3-selective modulator with anxiolytic effects, but the in vitro selectivity and the in vivo effects were discussed controversially in subsequent follow-up studies [93,94,95]. The synthesis of TP-003 (compound **87**) is described in WO 03/099816 and depicted in Scheme 18 [96,97]. 2-Bromo-5-Fluorobenzonitrile (compound **88**) is coupled with compound **89** to yield compound **90**. The addition of SnCl_2_ and sodium hydroxide in THF afforded compound **91**. Bromo-deamination led to the formation of compound **92**, which is transformed in compound **93** by treatment with bis(pinacolato)diboron. The following coupling led to the protected precursor (compound **95**), which was de-protected to obtain TP-003 (compound **87**).

**Figure 11:**
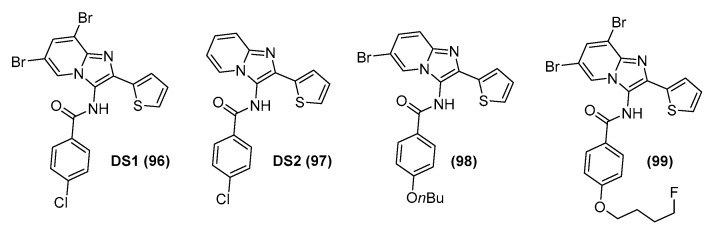
Structures of DS1, DS2, **98**, and **99**.

Among the imidazopyridines, not only ligands of the high-affinity binding site have been described. DS1 (compound **96**) and DS2 (compound **97**) have been introduced as somewhat δ-selective modulators of GABA_A_ receptors (Figure 11) [98,99]. δ-Subunits are present in certain extrasynaptic GABA_A_ receptors and contribute to pentamers devoid of high-affinity benzodiazepine binding sites [100,101].

Even though DS2 (compound **97**) cannot be considered fully δ selective [102], it shows a considerable functional preference for δ-containing GABA_A_ receptors and, therefore, different derivatives have been investigated. Among them, compound **98** and compound **99** (Figure 11) have shown promising pharmacological profiles with potential selectivity for α6β2/3δ GABA_A_ receptors, besides the δ selectivity, which is an interesting selectivity for the α6 subunit observed. 

Generally, the synthesis of such derivatives can be obtained in two steps [102] by reacting with an aromatic aldehyde with 2-amino pyridines in a multi-component fashion, which is followed by the formation of an amide bond.

**Scheme 19:**
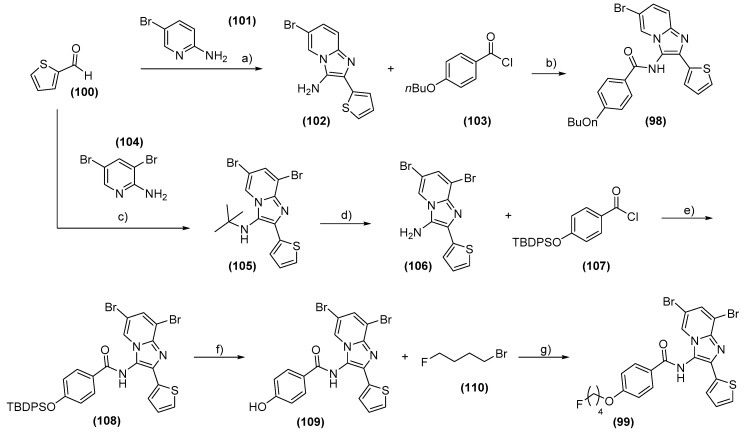
a): KCN, aq. Na_2_S_2_O_5_, 100 °C to 25 °C, 4 h, 56%, 3.5 h. b) Toluene/pyridine, 25 °C, 1 h, 57%. c) 2-isocyano-2-methylpropane, 1 M HClO_4_, MeOH, 25 °C, 24 h, 52%. d): 5 M HBr, 80–110 °C, 3.5 h, 98%. e) Toluene/pyridine, 25 °C, 1 h, 75%. f) TBAF, THF, 25 °C, 24 h, 88%, g) K_2_CO_3_, acetone, 60 °C, 48 h, 35%.

As shown in Scheme 19, compound **102** is obtained by the reaction of thiophene-2-carbaldehyde (compound **100**) with 2-amino-5-bromopyridine (compound **101**). The reaction of compound **102** with 4-butoxybenzoyl chloride yielded compound **98**. For the synthesis of compound **99**, thiophene-2-carbaldehyde (compound **100**) is reacted with 2-amino-3,5-dibromopyridine (compound **104**) in the presence of 2-isocyano-2-methylpropane to yield compound **105**. Upon treatment of compound **105** with HBr (5 M), intermediate **106** was formed. In the following step, the amide formation was performed via reaction with 4-((*tert*-butyldiphenylsilyl)oxy)benzoyl chloride (compound **107**). Deprotection with TBAF followed by a Williamson ether formation delivered compound **99** in a moderate yield (35%). These two syntheses already show that the substitution pattern on the core scaffold can be easily varied by using differently substituted starting materials and reagents.

#### 4.2.3. Fused Bicyclic GABA_A_ Receptor Modulators Containing Two Nitrogens and 6–7 Membered Rings 

##### Benzodiazepines

Benzodiazepines have dominated the scene of “tranquilizers” since the first representative chlordiazepoxide (Librium^®^, 1960) was introduced into the market [103]. Benzodiazepines are a large group of compounds widely used in clinical treatment. They are used for a range of different indications such as insomnia, anxiety, and depression, but also as anticonvulsants and anesthetics. Such a versatile use requires different characteristics. For example, when used in the treatment of anxiety or depression, the sedation and the hypnotic effect should be reduced, while, in procedural anesthesia, rapid sedation is highly desired. Pharmacologically, benzodiazepines can be classified according to their elimination half-life time in short-, intermediate-, and long-acting. Short-acting benzodiazepines, such as midazolam, are mostly used in anesthesia or insomnia due to their rapid onset. Long-acting benzodiazepines such as diazepam are preferred in the treatment of alcohol or drug withdrawal [104].

##### Classical Benzodiazepines

**Scheme 20:**
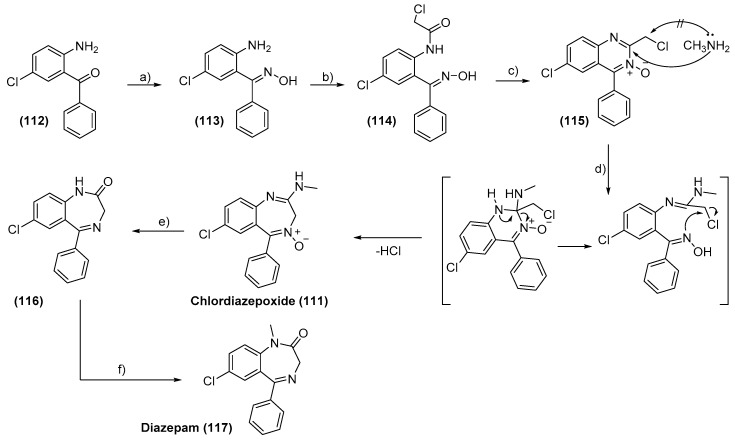
a) Hydroxylammonium chloride, EtOH, reflux, 8 h, 75%. b) chloroacetyl chloride, acetic acid, 50 °C, 10 min, 91%. c) NaOH, d) Methylamine, MeOH, rt, 3 d, 36%. e) H_2_O, f) 1. PCl_3_, 2. CH_3_I/base.

The first benzodiazepine, chlordiazepoxide (compound **111**), was synthesized serendipitously, as depicted in Scheme 20.

The addition of hydroxylamine to 2-amino-5-chlorobenzophenone (compound **112**) yielded oxime **113**, which, upon chloro-acetylation, yielded compound **114**. The cyclized benzodiazepine *N*-oxide (compound **115**) is formed by the treatment of **114** with NaOH. Interestingly, methylamine did not attack the methylene group carrying the Cl, but the equally electron-deficient C2-position of the heterocyclic core unexpectedly leading to compound **111**, via the proposed rearrangement mechanism displayed in Scheme 20. To further investigate the interesting sedative properties, many more compounds of the same class were then synthesized purposely. The synthetic routes via new synthetic approaches of selected examples are described in the following paragraphs.

**Scheme 21:**
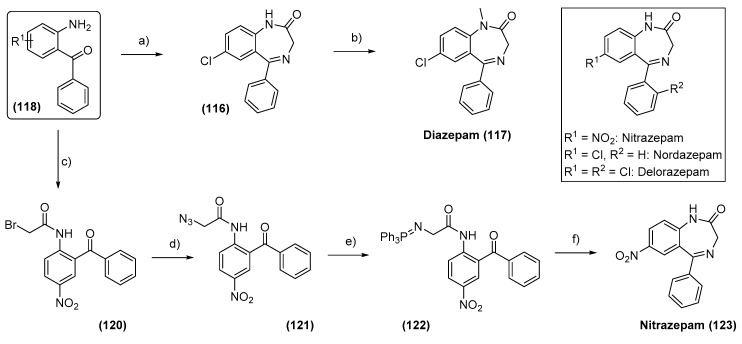
a) 1. Chloroacetyl chloride, toluene, rt, 2 h. 2. NH_4_OH/K_2_CO_3_, r.t, 3 h. b) dimethyl sulfate, K_2_CO_3_, r.t, 2 h. c) bromoacetyl bromide, toluene, reflux, 30 min, 88%. d) NaN_3_, EtOH, reflux, 30 min, 90%. e) PhP_3_, Toluene, rt. f) reflux, 1 h, 63%.

The structure of diazepam (compound **117**) can be obtained from the chlordiazepoxide **111** (Scheme 21) upon oxidative deamination, which is followed by a reduction of the *N*-oxide with PCl_3_ and *N*-methylation with methyl iodide [103].

Alternatively, diazepam can also be obtained starting from 2-amino-5-chlorobenzophenone (compound **112** or structure **118** R^1^: Cl) with chloroacetyl chloride and hydroxylamine, followed by a methylation (Scheme 21) [105]. Similar syntheses are used for nitrazepam (Mogadon ^®^), used as a hypnotic agent, delorazepam (En^®^), used as anxiolytic, and nordazepam (Nordaz^®^)(Scheme 21). An alternative synthesis for nitrazepam involves azide intermediate **121**, which is reacted with triphenylphosphine towards **122** including the substrate for cyclization to nitrazepam (compound **123**, Scheme 21) [106]. In principle, the same route was applied for the synthesis of the bromazepam (Lexotan^®^), which contains a 2-pyridyl ring instead of a phenyl ring in position 5 [107]. Additionally, substitution with different halogens on both rings of the benzophenones is reported. However, other substituents should, in principle, be tolerated. 

##### Acetylated Benzodiazepines

**Scheme 22:**
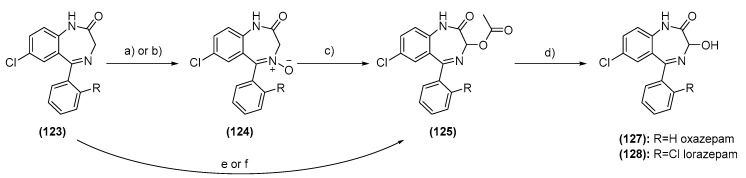
a) R = H: 30% H_2_O_2_, AcOH, Na_2_WO_4_ cat., 70–100 °C, 6 h, 84%. b) R = Cl, *m*-CPBA, DCM, rt, 10 h, 77%. c) R = H: acetic anhydride, 100 °C, 78%, 20 min or R = Cl: acetic anhydride, 70 °C; 79%, 3 h. d) NaOH, reflux. 80%. e) 1.1 eq. KI, 2 eq. Cu(Oac)_2_, 2 eq. KOAc, HOAc, 80 °C, 5 h (73%). f) 1 eq. I_2_, 2 eq. NH_4_S_2_O_8,_ 2 eq. KOAc, HOAc, 80 °C, 6 h (84%).

Position 3 of the general scaffold was modified to increase the polarity and, therefore, to increase the speed of metabolic elimination. A hydroxyl group is introduced in the case of lorazepam (compound **128**) (Tavor^®^) and oxazepam (compound **127**) (Alepam^®^, Murelax^®^, Serepax^®^). The general synthesis of these derivatives is depicted in Scheme 22 via an oxidation followed by an acetylation step [108,109,110]. Alternatively, acetylation using lead (IV) acetate and potassium iodide in acetic acid is reported (Scheme 22, conditions e and f) without the isolation of the iodinated intermediate [111,112].

##### Diazolobenzodiazepines

Among the different synthetic strategies developed after the discovery of the first benzodiazepines, the introduction of new rings annellated to the benzodiazepine scaffold was investigated. In 1982, midazolam (compound **129**) was marketed as Versed ^®^ and was initially used as a general anesthetic in procedural sedation and in the treatment of epileptic seizures due to its rapid onset [113]. Its water solubility makes it suitable for oral or buccal administration and, therefore, it makes it suitable to administer to children [114].

**Scheme 23:**
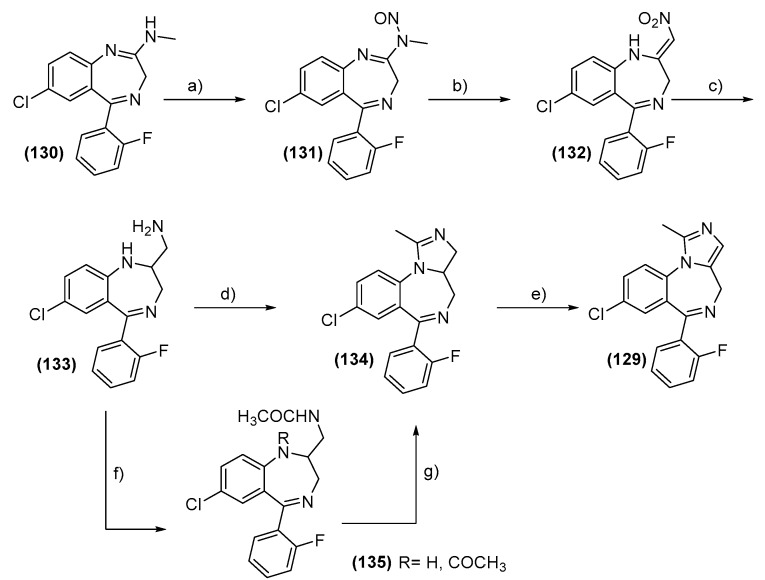
a) NaNO_2_, acetic acid, rt, 3 h, 60%. b) CH_3_NO_2_, Ko*t*Bu, DMF, rt, 1 h, 53%. c) Ni/H_2_, THF, 155 psi, 24 h, 83%. d) CH_3_C(Oet)_3_, xylene, reflux, 2 h, 68%. f) AC_2_O, pyridine, 100 °C, 4 h, 43%. g) PPA, 150–170 °C, 10 min., 23%. e) MnO_2_, toluene, reflux, 40 min., 58%.

The synthesis of midazolam (compound **129**) is achieved as depicted in Scheme 23. The nitrosation of amidine **130** afforded compound **131** in good yields. Compound **132** was obtained by the treatment of nitroso-amidine **131** with nitromethane in the presence of potassium *t*-butoxide. The hydrogenation over Raney nickel of compound **132** yielded compound **133** in high yields. Acetylation of compound **133** followed by a thermal cyclization delivered compound **134**. Alternatively, compound **134** could be obtained directly from **133** upon treatment with triethyl orthoacetate. In the last step, oxidation with MnO_2_ afforded compound **129 [113]**.

**Scheme 24:**
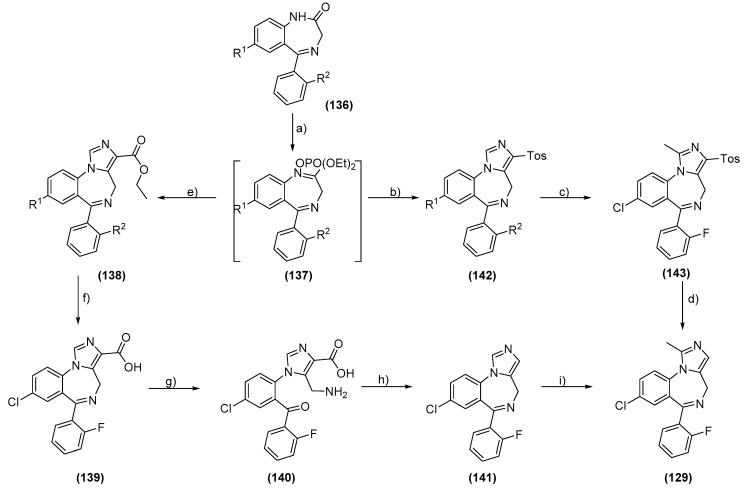
a) 1. Ko*t*Bu, THF, 0 °C. 2. ClPO(Oet)_2_, −20–0 °C. b) 1. TosMIC, 2. Ko*t*Bu, −20 °C–rt, 4 h, 80%. c) 1. BuLi, −78 °C 2. MeI, −78 °C–rt, 90%. d) Na-Hg amalgam, K_2_HPO_4_, EtOH/H_2_O, ultrasound, 72%. e) 1. CNCH_2_COOEt, 2. Ko*t*Bu, 4 h, 60%. f) NaOH, EtOH, rt, 4 h, 98%. g) HCl, EtOH, rt, 4 h, 83%. h) NMP, 200 °C, 2 h, 70%. i) 1. BuLi, −78 °C 2. MeI, −78 °C to rt, overnight, 90%.

In a different strategy, the methyl group can be inserted in position 2 of the imidazole via a lithiation, as outlined in Scheme 24. This method allows the insertion of different residues on the imidazole ring independently of the cyclization step [115]. 

##### Triazolobenzodiazepines

**Scheme 25:**
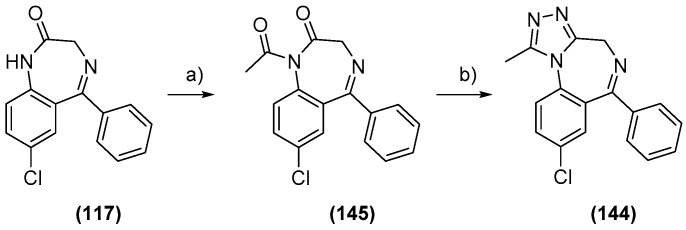
a) AC_2_O, K_2_CO_3_/KOH, r.t, 3 h, 80%. b) NH_2_NH_2_·H_2_O, NaOAc, reflux, 12 h, 75%.

Drugs containing a 1,3,4-triazole moiety, such as triazolam (Halcion ^®^), estazolam (Prosom^®^), and alprazolam (Xanax^®^) [116] were synthesized and introduced to the market to treat insomnia and panic attacks. The general synthesis of alprazolam (compound **144**) is depicted in Scheme 25 starting from diazepam (compound **117**) [105]. After the acetylation, the imide (compound **145**) is cyclized with hydrazine hydrate to the 1,3,4-triazole motif. Variation in the phenyl rings can again be obtained by using differently substituted benzophenones as starting materials for the benzodiazepine synthesis.

**Scheme 26:**
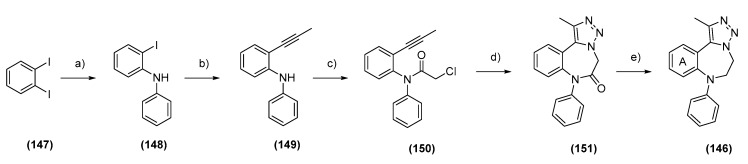
a) Aniline, Pd(dba)_2,_ Xantphos, Cs_2_CO_3_, toluene, 110 °C, overnight, 27%. b) trimethyl(prop-1-yn-1-yl)silane, TBAF, CuI, Pd(PPh_3_)_4_, THF, 70 °C, overnight, 84%; c) 2-chloroacetyl chloride, K_2_CO_3_, DCM, 45 °C, overnight, 64%. d) NaN_3_, DMF, 150 °C, 2 h, 13%. e) BH_3_, THF, 70 °C, 2 h, 88%.

The synthesis of a 1,2,3-triazolobenzodiazepine scaffold (structure 146) is outlined in Scheme 26. Compound **148** was obtained via a Buchwald-Hartwig coupling from 1,2-diiodobenzene (compound **147**) and aniline. Subsequent Sonogashira coupling, which was followed by acetylation, yielded compound **115** in low yields. Even though this step represents a drawback of this synthesis, for similar derivatives (e.g., with a bromine atom in position 9), better yields could be achieved with the same conditions. Lastly, a cyclo-addition with sodium azide and a carbonyl reduction with BH_3_ were performed to obtain the triazolobenzodiadepine scaffold **146** [117]. The cyclo-addition in this specific example gave only a poor yield of 13% due to unoptimized reaction conditions.

**Scheme 27:**
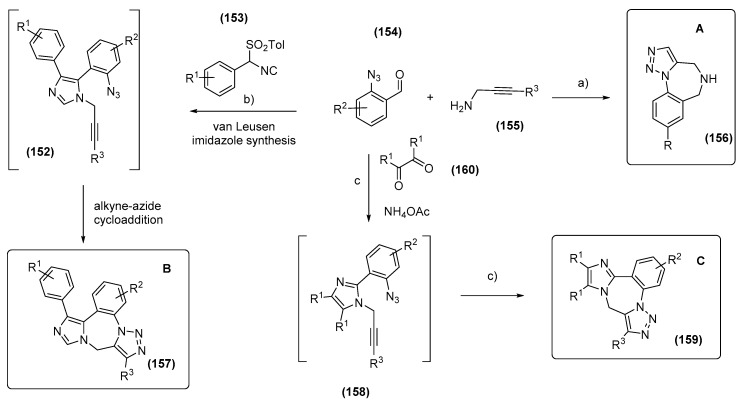
a) 1. AcOH, NaB(Oac)_3_H, DCE, rt, 3 h. 2. Toluene, 100 °C, 4.5 h. b) 1. Amine and aldehyde, DMF, rt, 2.5 h, 2. TosMIC, K_2_CO_3_, rt, 17 h. c) InCl_3,_ (10 mol%), MeOH, 60–100 °C, 10–72 h.

Differently substituted anilines are tolerated, such as *p*-chloroaniline or *m*-bromoaniline. Additionally, a slightly modified procedure starting from substituted 2-iodoanilines (R = Br, COOMe) instead of 1,2-diiodobenzene, allows the introduction of substituents on ring A of the benzodiazepine scaffold (scheme not shown). These bromo or ester derivatives can be used for further modifications (e.g., with coupling reactions). Furthermore, in the last few years, multi-component reactions have been reported for the synthesis of various triazolobenzodiazepine scaffolds (A, B, C), which deliver compound libraries quickly (Scheme 27). Multi-component reactions involving aldehydes, amines, and acylating agents are a suitable and versatile strategy to access polycyclic heterocyclic scaffolds [118]. In particular, structures containing both the 1,4 benzodiazepine ring system and the triazole system are of high interest because of their medical potential [119]. All three strategies require a 2-azidobenzaldehyde (structure **154**) and a propargylamine derivative (structure **154**) as key compounds with the addition of other reagents.

For example, Donald et al. [120,121] demonstrated that the triazolobenzodiazepine scaffold (structure **156**) is easily accessible via a reductive amination and an azide-alkyne cycloaddition as shown in Scheme 27 (route A). The scaffold obtained can be further modified to rapidly access a variety of compounds with different *N*-substituents. 

Another azide-alkyne cycloaddition was investigated by Djuric et al. as a modification of the van Leusen imidazole synthesis for the synthesis of imidazole and triazole-fused benzodiazepines [122]. As shown in Scheme 27, after the formation of the imidazole (structure **152**), an intramolecular cycloaddition is performed. The different fused triazole benzodiazepines accessible with this route have the general formula **157**, with R^1^ being -H or F-, R^2^ being H- or dioxolane and R^3^ being either -H, -Me or -Ph.

A third variation was reported by Nguyen et al. [123] (Scheme 27C) when taking advantage of an intramolecular 1,3-dipolar Huisgen cyclo-addition. The triazolobenzodiazepine scaffold is obtained in a one-pot reaction using a 1,2-diketone (structure **160**), an azido-aldehyde (structure **154**), a propargylamine (structure **155**), and an ammonia reactant in the presence of a Lewis acid. This method tolerates a variety of 1,2-diketones (R^1^: Ph, CH_3_, H, *p*-BrPh, *p*-MeOPh, *p*-MePh) as well as different azo-benzaldehydes (R^2^: CO_2_Me, Br, dioxolane and -H) and propargylic amines (R^3^: H, Et, Ph) with a yield range of 18–72%.

**Scheme 28:**
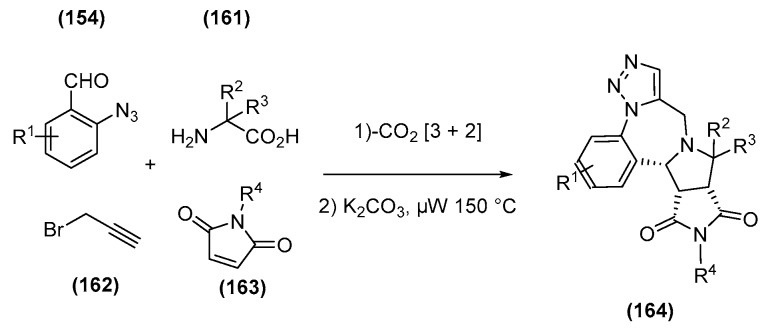
1. AcOH, CH_3_CN, 110 °C 6 h. 2. K_2_CO_3_, µw, 1 h, 35–65%.

Recently, a one-pot synthesis of triazolobenzodiazepines (structure **164**) has been accomplished using a [3 + 2] cycloaddition of azomethine yield and *N*-ethylmaleimide (structure **163**), which is followed by an *N*-propargylation and a click reaction [124] (Scheme 28). The method accommodates the use of readily available amino acids (R^2^ and R^3^ = CH_3_ or R^2^ = H and R^3^ = iPr. Or Ph), different azido-aldehydes (R^1^ = H, CF_3_, Br, Cl), and different maleimides (R^4^ = Et, Me, Bn, Ph), which generates as a byproduct of only H_2_O and CO_2_.

#### 4.2.4. Fused Bicyclic GABA_A_ Receptor Modulators Containing Three Nitrogens 

##### Pyrazolopyrimidine

**Scheme 29:**
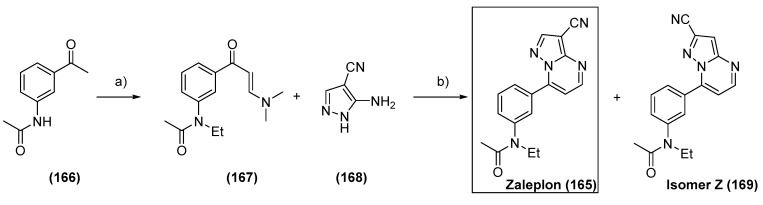
a) 1. DMF-DMAc, reflux, 8 h, 2. NaH, EtBr, rt, 0.5 h, yield not reported, b) 30% acetic acid in H_2_O, 50 °C, 2 h, 86%.

Zaleplon (compound **165**), also belonging to the Z-drugs, is a pyrazolopyrimidine used as a short-acting hypnotic [125,126]. The synthesis of zaleplon (compound **165**) is performed as outlined in Scheme 29 with the reaction of acetamide 167 with 5-amino-1*H*-pyrazole-4-carbonitrile (compound **168**. One major drawback of the synthesis of zaleplon (compound **165**) is the formation of the isomer Z (compound **169**) as a side-product. Different acidic conditions are reported. Aqueous acetic or formic acids are preferred over anhydrous acetic acid, which leads to the formation of a high amount of the desired isomer (compound **165**) [89,127].

**Scheme 30:**
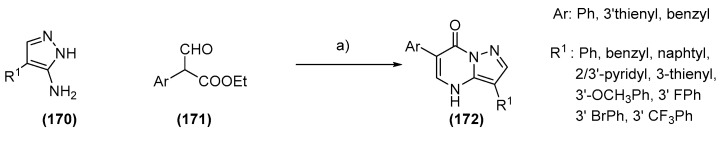
Reflux, diglyme/ethylene glycol, 8 h, yield not reported.

Among the pyrazolopyrimidines investigated as sedative-hypnotic agents, Indiplon [128] received an initial approval letter from the United States Food and Drug Administration (FDA). However, after further investigations, it remained unapproved unless more clinical studies are conducted. Other structures containing a pyrazolopyrimidine core (structure **172**) were investigated as potential ligands for the GABA_A_ receptors [129,130]. One compound of this class (Ar: 3′-thienyl, R^1^: 2′-pyridyl) showed a promising selectivity for α1 containing receptors [129]. These structures can be obtained in a one-step reaction as displayed in general Scheme 30, which allows a rapid synthesis of a variety of derivatives [131].

##### Cyclopyrrolones

**Scheme 31:**
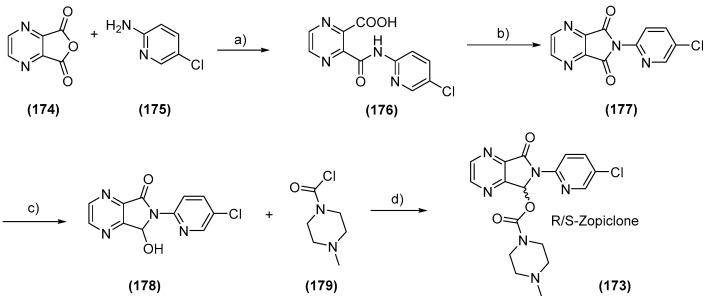
a) I, reflux, 1.5 h. b) SOCl_2_, reflux. c) KBH_4_, dioxane/water, 13 °C, 6 min. d) base (NaH. K_2_CO_3_, Et_3_N, DBU), solvent (DMF, DCM, benzene, 50 °C).

The last Z-drug presented here is Zopiclone (compound **173**) [132], which is commercially available in Europe as a racemic mixture (Imovane ^®^), while, in the USA, the active *S*-enantiomer (compound **173**) [133] is marketed as Lunesta ^®^ in an optically pure form (Scheme 31). Like the two other drugs in this class, it is prescribed for the treatment of insomnia. Chemically, zopiclone belongs to the class of cyclopyrrolones.

The synthesis of zopiclone is performed via the reaction of compound **178** and compound **179** or its hydrochloride in the presence of a base [134,135,136] (Scheme 31). The precursor (alcohol **178**) can be prepared by starting from the reaction between compound **174** and compound **175**. The resulting acid is refluxed in thionyl chloride to yield compound **177**. The latter upon addition of potassium borohydride in an aqueous solution is converted to compound **178**. Final esterification delivers the target *R/S*-Zopiclone (compound **173**).

Alternatively, compound **177** can be synthesized starting from pyrazine-2,3-dicarboxylic acid and 5-chloropyridine-2-amine in a one-step conversion in acetic anhydride (scheme not shown) [137]. 

#### 4.2.5. Fused Bicyclic GABA_A_ Receptor Modulators Containing Four Nitrogens

##### Pyrazolotriazines

**Figure 12:**
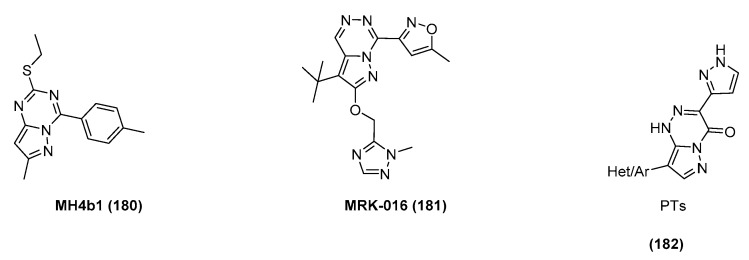
GABA_A_ receptor ligands containing the pyrazolotriazine scaffold.

Different structures are reported as being active on GABA_A_ receptors with a pyrazolotriazine (PT) scaffold (Figure 12).

**Scheme 32:**
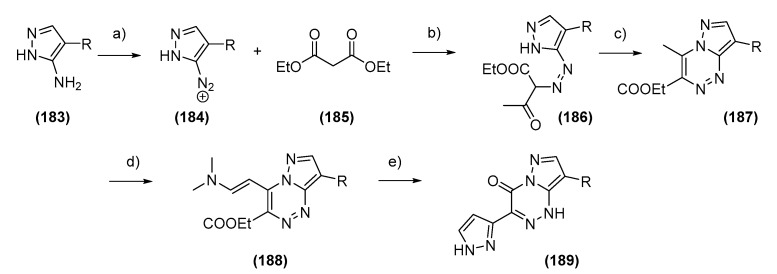
a) HCl, NaNO_2_/H_2_O, rt. b) NaOAc, ethyl acetoacetate, EtOH, rt, 1 h. c) DMF-DMAc, toluene, piperidine cat., 90 °C. d) hydrazine hydrate 60%, acetic acid, sodium acetate, reflux.

PT structures were synthesized and investigated as aza-isomers of pyrazolopyrimidines [138]. Pyrazolotriazines are prepared starting from differently substituted 3-aminopyrazoles (structure **183**) (Scheme 32). A diazotization to structure **184** followed by azo-coupling to structure **186** and a cyclization with ethyl acetoacetate yielded 8-substituted structure **187**. Compounds of general structure **187** were then further transformed into structure **188** with a mixture of dimethylformamide-dimethylacetamide (DMF-DMAc). Lastly, the addition of hydrazine hydrate in the presence of sodium acetate in acetic acid led to structure **189**. This synthesis could be used for a broad range of substituted aminopyrazoles with R being 2- or 3-pyridyl, 2-thienyl, 1- or 2-naphthyl and substituted aryl rings carrying electron-donating or withdrawing substituents.

Compounds with structure **189** were found to have a K_i_ in the nM range for the benzodiazepine binding site. The product with R = 3-trifluoromethylphenyl showed selectivity towards α1. Of the same class of compounds, MRK-016 (compound **181**) [139] is reported to be an “α5-selective inverse agonist,” which is the historical term for a negative allosteric modulator at the benzodiazepine binding site of receptors that contain α5 subunits. 

**Scheme 33:**
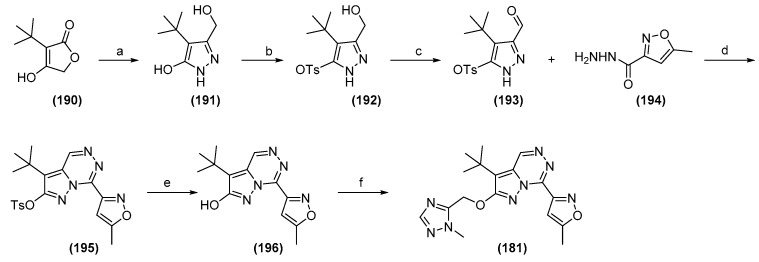
a) NH_2_NH_2_‚H_2_O, EtOH, reflux, quant. b) *p*-toluenesulfonyl chloride, Et_3_N, DCM, 67%. c) MnO_2_, CHCl_3_, reflux, 95%. d) Xylene, reflux, 67%. e) 4 N NaOH, MeOH, 65 °C, 85%. f) K_2_CO_3_, DMF, 5-chloromethyl-1-methyl-1*H*-1,2,4-triazole, 76%.

The synthesis of this compound is described by Chambers et al. [140]. Compound **190** was heated with hydrazine hydrate to yield pyrazole **191**. After protecting the hydroxyl moiety, the primary alcohol of structure **192** was oxidized to aldehyde **193**. Condensation between the aldehyde **193** and the isoxazole **194** afforded compound **195**. After deprotection, compound **196** was reacted with 5-chloromethyl-1-methyl-1*H*-1,2,4-triazole to yield MRK-016 (compound **181**) (Scheme 33). 

##### Triazolopyridazines

**Figure 13:**
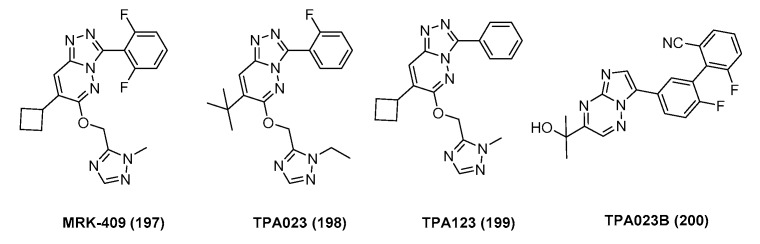
Structure of most studied triazolopyridazine derivatives.

Triazolopyridazines are a scaffold from which compounds MRK-409 (compound **197**), TPA023 (compound **198**), and TPA023B (compound **200**) have progressed into clinical trials. This scaffold was developed under the controversially debated hypothesis that compounds that show a functional preference toward the α2- or α3-subunits of the GABA_A_ receptors should have selective anxiolytic effects [141,142]. The structures of some highlighted derivatives of this family of compounds are shown in Figure 13.

**Scheme 34:**
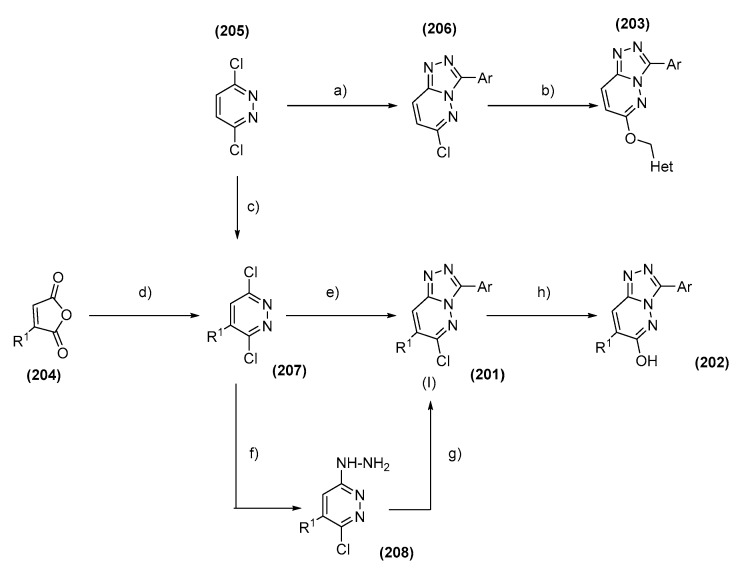
a) Ar-CO-NH-NH_2_, TEA, xylene or 1,4-dioxane, reflux. b) Het-CH_2_-OH, Base (NaH or LiHMDS), DMf. c) (NH_4_)_2_S_2_O_8_, AgNO_3_, R_1_-COOH, H_2_SO_4_, H_2_O, 70 °C. d) 1. NH_2_-NH_2_·H_2_O, AcOH, NaOAc, reflux. 2. POCl_3_. e) Ar-CO-NH-NH_2_, TEA, xylene or 1,4-dioxane, reflux. f) NH_2_-NH_2_, solvent not reported. g) Ar-CO-Cl, solvent not reported. h) NaOH, 1,4-dioxane, reflux.

Synthesis of this scaffold (structure **204**) has been reported from three different precursors (structures **201**, **202**, and **203**) for the final step. The synthesis of intermediates **201**, **202**, and **203** is depicted in Scheme 34 and it begins with 3,6-dichloropyridazine (compound **205**), which can be treated with the desired hydrazide and further cyclized in one step to obtain the accordingly substituted triazolopyridazine scaffold (structure **206**). The desired ether in position 6 can be easily prepared using Williamson etherification conditions. Additionally, a substituent of choice can be introduced to the initial 3,6-dichloropyridazine (compound **207**) using either a homolytic alkylation in the heteroaromatic ring or by synthesizing the pyridazine using the substituted maleic anhydride (structure **204**) as a source of the required 1,4-diketone, hydrazine, and subsequent halogenation with phosphorus oxychloride. Afterward, the substituted dichloropyridazine (structure **207**) can be treated again either with the same conditions mentioned above to obtain the triazolopyridazine moiety or an alternative way in a stepwise manner, using hydrazine and the corresponding acyl chloride. Lastly, intermediate **202** can be obtained from **201** just by refluxing it with sodium hydroxide in the presence of 1,4-dioxane [143,144,145]. 

**Scheme 35:**
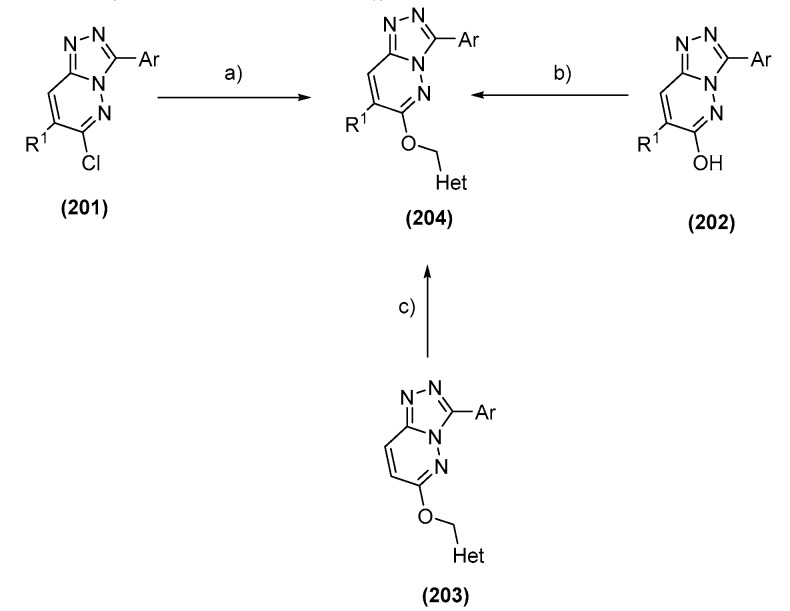
a) Het-CH_2_-OH, base (NaH or LiHMDS), DMf. b) Het-CH_2_-Cl, base (NaH or LiHMDS), DMf. c) (NH_4_)_2_S_2_O_8_, AgNO_3_, R_1_-COOH, acidic conditions (H_2_SO_4_ aq, CH_3_CN aq), reflux.

Intermediates **201-203** are then converted to the target scaffold **204** via the three methods shown in Scheme 35. Conditions a) and b) correspond to a classical Williamson etherification protocol, where either the necessary alcohol or the halide is attached to the main heterocyclic core. The third reported strategy was performed by a homolytic alkylation of the heteroaromatic ring, generating the required alkyl radical from a silver-catalyzed decarboxylation of the corresponding carboxylic acid using ammonium persulphate. The synthesis of the required intermediates **201-203** is shown in Scheme 35. 

### 4.3. Fused Tricyclic GABA_A_ Receptor Modulators 

#### 4.3.1. Fused Tricyclic GABA_A_ Receptor Modulators Containing Two Nitrogens

##### β-Carbolines

**Figure 14:**
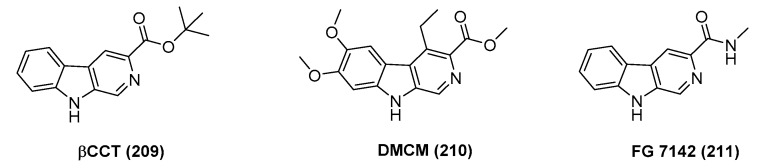
Chemical structure of carbolines as GABA_A_ modulators.

β-Carbolines were identified in the 1980s as potential endogenous ligands for the benzodiazepine binding site (Figure 14). In this versatile scaffold, compounds with diverse profiles ranging from strong to moderate unselective negative modulation DMCM (compound **210**) and FG-7142 (compound **211**) to mixed NAM/weak PAM subtype profiles (β-carboline-3-carboxylate-*t*-butyl ester, βCCt) were identified, which makes them heavily used research tools in rodent studies [141,142,143,144].

**Scheme 36:**
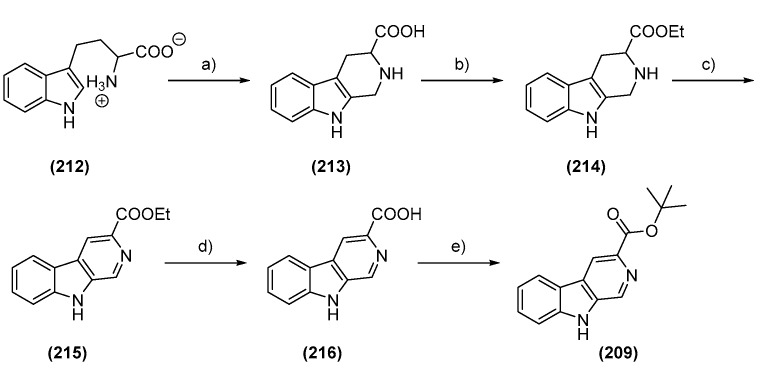
a) HCHO, NaOH, 37 °C, 95%. b) EtOH, 2 eq H_2_SO_4_, reflux, 83%. c) MnO_2_, benzene, reflux, 80%. d) NaOH, H_2_O, reflux, 90%. e) CDI/DMF, DBU, 50 eq. *t*-BuOH, 85 °C, 75%.

The synthetic accessibility of derivatives such as βCCT (compound **209**) is outlined in Scheme 36. As the first step, tetrahydro-β-carboline (compound **213**) was obtained from d,l-tryptophan (compound **212**) via a Pictet–Spengler reaction on a kilogram scale. The tetrahydro-β-carboline (compound **213**) underwent a Fischer esterification, and, afterward, oxidation with activated MnO_2_, which yielded the product βCCE (compound **215**). The ester was further hydrolyzed and the corresponding acid re-esterified with *t*-butanol and CDI to yield βCCT (compound **209**) [146]. Compound FG 7142 (compound **211**) can be easily achieved by treating intermediate (compound **216**) with methylamine overnight, which yields the product in a period of 12 h.

**Scheme 37:**
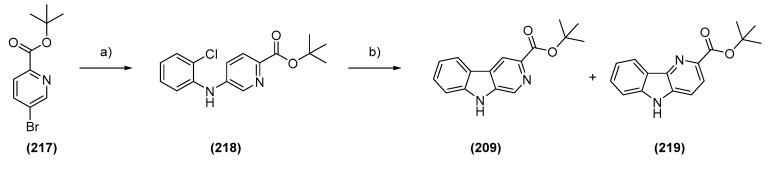
a) 2-chloroaniline, 5 mol% Pd(OAC)_2_, 7.5 mol% Xantphos, 1.5 eq. Cs_2_CO_3_, toluene, 100 °C, 15 h, 94%. b) Pd(OAC)_2_, (*t*-Bu)_3_P HBF_4_, K_2_CO_3_, DMAc, 120 °C, 16 h, 83%.

Alternatively, an improved large-scale two-step route for carboline βCCT was developed by performing a Buchwald-Hartwig coupling of *t*-butyl 5-bromopicolinate (compound **217**) and 2-chloroaniline. The resulting diarylamine (compound **218**) underwent an intramolecular Heck cyclization, which was performed on a 10-g scale and afforded a mixture of βCCT (compound **209**) and its regioisomer, δCCT (compound **219**), in a ratio 2:1, respectively. Despite this, overall yields improved significantly, from the previous 35% to 52% on a 10-g scale and authors declared that the separation of regioisomers was performed with simple flash chromatography (Scheme 37). This protocol was also applied to derivatives with different alkoxy substituents ortho to the pyridyl nitrogen atom, which yielded the desired coupling product in high yields. When the subsequent Heck cyclization was performed, the ratios between ß-carbolines and δ-carbolines seemed to be influenced by the substituents located ortho to the pyridyl nitrogen atom. Smaller substituents provided equimolar amounts of both isomers, but bulkier substituents (*i*-Pr, *t*-Bu) afforded β-carbolines as the main product (scheme not shown) [147]. 

#### 4.3.2. Fused Tricyclic GABA_A_ Receptor Modulators Containing Three Nitrogens

##### Pyrazoloquinolinones

Pyrazoloquinolinones (PQs) (structure **220**) were reported for the first time as ligands of the “benzodiazepine receptor” (which is a historical term for the high-affinity benzodiazepine sites present at GABA_A_ receptors) in 1982 [148] when the synthesis of three PQs was reported, and these derivatives were presented as useful compounds to study the complex pharmacology of this receptor. The three compounds, in fact, showed three different in vivo pharmacological behaviors, which were ascribed to the benzodiazepine binding site. While the unsubstituted derivative, CGS8216 (compound **225**), showed benzodiazepine-antagonist properties, the *p*-chloro derivative (CGS9896) (compound **226**) showed benzodiazepine agonistic properties and the *p*-methoxy derivative (CGS9895) (compound **227**) behaved consistently with benzodiazepine partial agonists. Among these three, the agonist CGS9896 (compound **226**) (*p*-chloro) was claimed to be a safe anti-anxiety agent due to the lack of neurological deficits and its non-sedative behavior. Furthermore, functional studies revealed that PQs interact with additional sites of GABA_A_ receptors [149], which makes it impossible to assign their in vivo effects of any single binding site. 

**Scheme 38:**
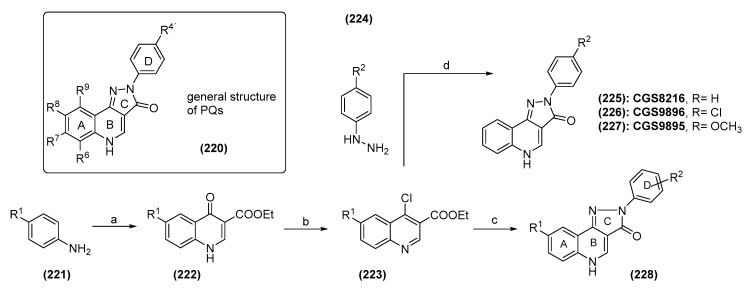
a) DEEMM, Ph_2_O, reflux. b) POCl_3_, reflux. c) arylhydrazine, DMAc, 140 °C. d) xylene, 120 °C.

The synthetic strategy for the synthesis of PQ derivatives remained unchanged with few modifications and still follows the route displayed in Scheme 38. The quinoline derivative (structure **222**) is obtained starting from the corresponding *p*-anisidine (structure **221**) upon condensation with diethyl ethoxymethylenemalonate (DEEMM), which was followed by a thermal cyclization. The chlorination of the quinoline derivative with POCl_3_ (ii) yields the precursor of the PQ (structure **223**). The required phenylhydrazine (structure **224**) is used for the formation of PQs with different substituents in the D ring. Different A-ring substitution patterns are obtained using different aniline derivatives as starting materials.

**Scheme 39:**
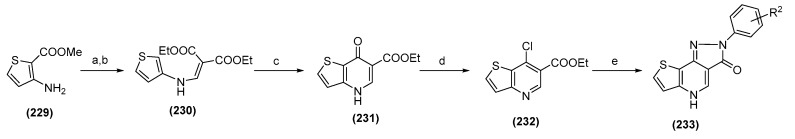
a) 1 M, NaOH, reflux. b) DEEMM, AcOH, toluene, reflux. c) Ph_2_O, reflux. d) POCl_3_, reflux. e) arylhydrazine, DMAc, 140 °C.

Replacing the A-ring with electron-rich heterocyclic scaffolds (structure **223**) requires modification of the synthetic sequence (Scheme 39). Thiophene-3-amine would be too electron-rich to be a stable starting material. Hence, compound **229** is used as starting material and in situ hydrolyzed, decarboxylated, and reacted with DEEMM as reported in the supplementary information of Varagic et al. [149]. From then on, the sequence follows the same steps as with carbocyclic A-rings.

Due to the inconsistent results obtained during the investigation of their potential anxiolytic effect, PQs were not further investigated as potential therapeutic agents [150,151], but they kept being used as pharmacological tools in the studies of GABA_A_ receptors. 

Over the years, the general scaffold of PQ has been extensively modified, especially on ring A and ring D [152,153,154], which results in compounds with different pharmacological profiles [155,156]. Additionally, studies on the binding site of PQs were performed. While displaying a higher affinity for the BZD-site, PQs such as CGS9895 (compound **227**) were reported to have a modulatory effect at a binding site situated between the α+/β− interfaces, where it displays a lower potency compared to the BZD-site [19,157]. Replacement of ring A with an aliphatic ring resulted in a null modulator at the benzodiazepine (BZD) binding site, and the replacement with a thiophene ring led to the synthesis of a non-binder [149]. Additionally, the relationship between the substitution pattern and binding activity was investigated. It was demonstrated that substituents in positions 7 and 9 caused a lack of affinity at the BDZ site, while substitution in position 8 associated with a substituent on the phenyl ring on *N*2 increased the activity [156]. 

#### 4.3.3. Fused Tricyclic GABA_A_ Receptor Modulators Containing Four Nitrogens

##### Triazoloquinazolines

**Figure 15:**
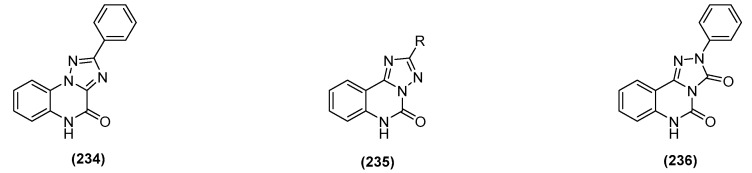
Triazoloquinazolines.

After the discovery of PQ derivatives (structure **220**), and their modulatory activity, more compounds with heterotricyclic structures became appealing chemotypes. The replacement of ring C with a triazole led to structure **234** (Figure 15). However, it did not lead to an improvement in the activity in comparison with the PQ scaffold. The only exception was the ortho fluorophenyl, which had a comparable activity [158,159]. 

**Scheme 40:**
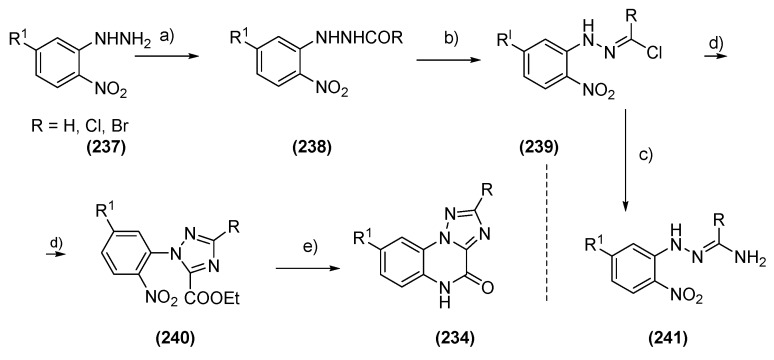
a) RCOCl, EtOH/pyridine, reflux, 1 h. b) POCl_3_, reflux, 1 h. c) NH_3_ (g), d) CICOCOOEI. (e) Fe/CH_3_COOH.

Triazole containing structures, such as compound **235** (Figure 15), were initially reported as antagonists or partial inverse agonists for the BZR (the historical term for the high-affinity benzodiazepine binding sites) [160]. The synthesis of such compounds is achieved by starting from the arylhydrazines (structure **237**) upon treatment with aryl chlorides, which was followed by a chlorination. Structure **239** was then treated with ammonia to yield structure **241**. The reaction of the latter with ethyl oxalyl chloride led directly or upon further heating to structure **240**. In the last step, a reduction performed with Fe and acetic acid led to the final structure **235** (Scheme 40) [161].

**Figure 16:**
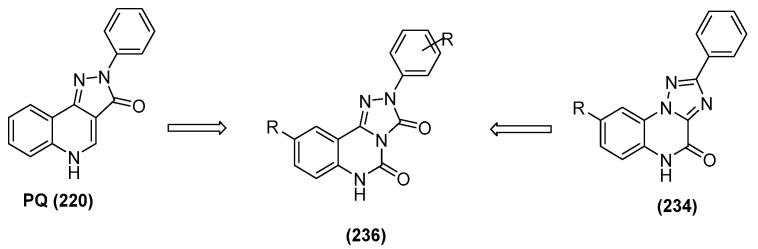
Design of 2-Aryltriazoloquinazolinediones.

Looking at the structures of the GABA_A_ receptors ligands, PQs, and phenyltriazoloquinoxalone (structure **234**), the design of triazoloquinazolinediones (structure **236**) was the logical next step (Figure 16) [162].

**Scheme 41:**
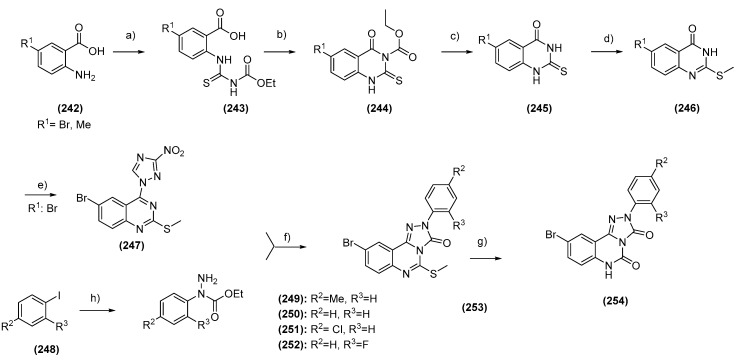
a) Ethoxycarbonyl isothiocyanate, MeCN, 80 °C, 4 h, 91–95%. b) Ac_2_O, 60 °C, 1 h, 93–100%. c) 0.5 M NaOMe in MeOH, THF, reflux, 90 min, 88–100%. d) DMF, 0.5 M NaOMe in MeOH, then MeI, rt, 10 h 77–90%. e) 3-nitro-1*H*-1,2,4-triazole, PPh_3_, I_2_, toluene, DIPEA, 95 °C, 1 h, 89%. f) DIPEA, 249-252, 110 °C, 60 h, 51–70%. g) *m*-CPBA (77%), CH_2_Cl_2_, rt, 6-h quantitative yield. h) CuI, 1,10-phenantrolein, ethyl carbazate, Cs_2_CO_3_, DMF, 80 °C, 16 h, yields compound 249: 83% compound 250: 76%, compound 251: 40%, compound 252: 22%.

According to pharmacophore studies, this type of structure fulfills the requirements for a strong affinity to the benzodiazepine binding site [163]. The synthesis of triazoloquinazolinedione (structure **254**) was achieved starting with the anthranilic acid derivative (structure **242**), which was treated with ethoxycarbonyl isothiocyanate. This is followed by a cyclization in acetic anhydride (structure **244**). Deprotection and methylation yielded the precursor (structure **246**), which was treated with 3-nitro-1*H*-1,2,4-triazole to yield structure **247**. The reaction between structure **247** and compounds **249**-**252** delivered (structure **243**). Triazoloquinazolinediones (structure **254**) are then obtained upon treatment of (structure **243**) with *m*-CPBA (Scheme 41).

**Scheme 42:**
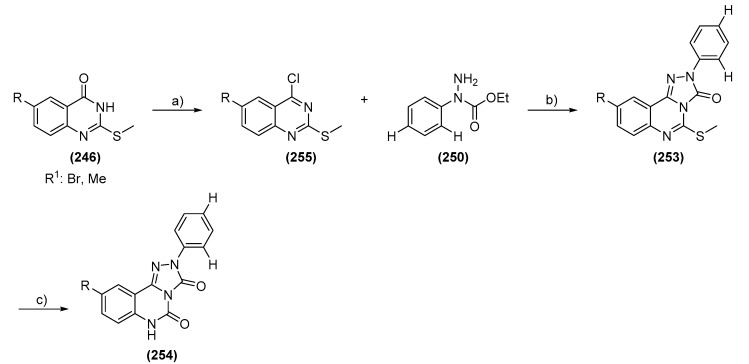
a) POCl_3_, pyridine, 110 °C, 18 h, 75–77%. b) DIPEA, 110 °C, 60 h, 71–75%. c) *m*-CPBA (77%), CH_2_Cl_2_, rt, 6 h, quantitative yields.

Alternatively, structure **246** can be activated with phosphorous oxychloride before the addition of compound **250** to yield structure **253** with yields comparable with the nitrotriazole strategy (Scheme 42).

**Scheme 43:**
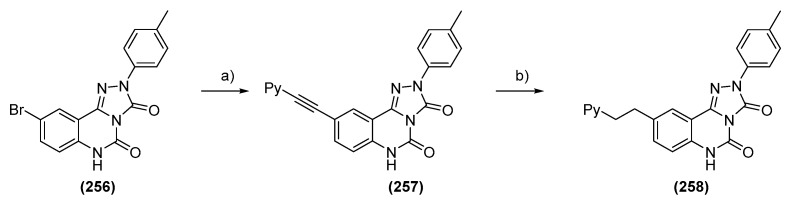
a) Pd(OAC)_2_, PPh_3_, RcCH, NEt_3_, DMF, 100 °C, 18 h, 50%. b) H_2_ (1 atm) 10% Pd/C, CH_2_Cl_2_, MeOH, rt, 18 h, 91%.

The compounds of this class display good affinities for the BZD site, determined in vitro by displacement of ^3^H-Flumazenil in rat cortical tissue, according to the pharmacophore model. Compound **256** can be further modified with cross-coupling reactions. For example, copper-free Sonogashira coupling conditions followed by hydrogenation (Scheme 43) are used for the synthesis of **258**, which displayed high-affinity and a certain subtype selectivity for α1ß3γ2 GABA_A_ receptors.

##### Pyrazolobenzotriazines

The pyrazolobenzotriazine scaffold was first reported as a GABA_A_ receptor ligand in 1998 [164]. During the past 10 years, *N*-oxide derivatives of this scaffold were synthesized and studied both in vitro and in vivo with some derivatives having binding affinities to the benzodiazepine binding site lower than 10 nM [165,166]. Some of them possess anxiolytic activity [166], which, together, suggests positive modulatory effects elicited via the benzodiazepine binding site. 

**Scheme 44:**
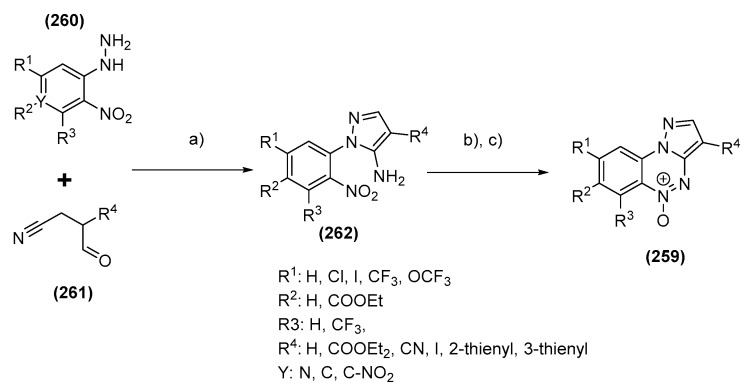
a) EtOH. b) H_2_SO_4_. c) 10% aq NaOH.

There are several strategies reported to access this scaffold. An example of how to synthetically access the main scaffold is shown in Scheme 44. The accordingly substituted 2-nitrophenylhydrazine (structure **260**) was reacted with 2-oxosuccinonitrile (structure **261**) to obtain the corresponding 2-(2-nitrophenyl)-5-aminopyrazole-4-acetonitrile (structure **262**). Acidic hydrolysis of this compound gave, as a result, the corresponding acetamide derivative, and further treatment in alkaline media-initiated cyclization to the benzotriazine system (structure **259**).

Pyrazolobenzotriazine (structure **259**) derivatives containing halogenated substituents were useful building blocks to perform subsequent Suzuki-Miyaura cross-coupling reactions with several aryl or heteroaryl boronic acids by incorporating these cyclic substituents into the main core. Further modifications can be performed as well to the R^4^ substituents in order for convenient functional groups such as carboxylic acids, amides, or other functional groups to introduce several heterocyclic five-membered rings (Scheme not shown). 

## 5. Conclusions

It was shown in the previous sections that a large number of heterocyclic scaffolds are, or have been, under investigation as GABA_A_ receptor modulators. It can be observed that the number of examples for a given scaffold that underwent biological testing is directly proportional to the ease of access of the corresponding structures. However, focusing on easily accessible chemotypes is not how medicinal chemistry will experience progress in the future. Hence, it is the task of synthetic chemists to develop methods to make any scaffold accessible within only a few synthetic steps and to develop the potential to deliver compounds with good diversity regarding substitution patterns. Thus, there is still an infinite playground to develop new and exciting heterocyclic chemistry in order to enable the discovery of improved or completely new biological activity, especially within the pending challenges in GABA_A_ pharmacology toward improved subtype-selective compounds. New (or re-discovered) developments in synthesis will be important contributors to reach this goal. In particular, it will be interesting to see how a broader implementation of biocatalysis, electrochemistry, continuous-flow chemistry, and photo(redox)chemistry will impact drug development in the future.

## Abbreviations

ADME	Absorption, Distribution, Metabolism, and Excretion
ACN	Acetonitrile
Bbt	Barbiturate
BZD	Benzodiazepine(s)
BZR	Benzodiazepine receptor
CDI	1,1′-Carbonyldiimidazole
DBU	1,8-Diazabicyclo[5.4.0]undec-7-ene
DCC	*N,N′*-Dicyclohexylcarbodiimide
DCE	1,2-dichloroethane
DCM	Dichloromethane
DMAc	Dimethylacetamide
DMAP	4-Dimethylaminopyridine
DMF	Dimethylformamide
DMSO	Dimethyl sulfoxide
DNA	Deoxyribonucleic acid
DEEMM	Diethyl ethoxymethylenemalonate
DIPEA	*N,N*-Diisopropylethylamine
EBOB	Ethynylbicycloorthobenzoate
ECD	Extracellular domain
Eto	Etomidate
GABA	Gamma-aminobutyric acid
GABA_A_	GABA_A_ receptors
HATU	Hexafluorophosphate Azabenzotriazole Tetramethyl Uronium
HTIB	[Hydroxy(tosyloxy)iodo]benzene
IPA HCl	Isopropanol hydrochloride
KOtBu	Potassium *tert*-butoxide
NAM	Negative Allosteric Modulator
NMP	*N*-Methyl-2-Pyrrolidone
OTC	Over-the-counter
PAM	Positive Allosteric Modulator
PET	Positron Emmission Tomography
PQ	Pyrazoloquinolinone
SAM	Silent Allosteric Modulator
TBBAB	Tetra-*n*-butylammonium bromide
TBAF	Tetrabutylammonium fluoride
TEA	Triethylamine
THF	Tetrahydrofuran
TMD	Transmembrane domain
TMP	2,2,6,6-Tetramethylpiperidine
TosMIC	*p*-Toluenesulfonylmethyl isocyanide
